# Chemical Profiles of the Volatilome and Fatty Acids of “Suero Costeño” (Fermented Cream)/Raw Milk from Colombia: Promising Criteria for the Autochthonous-Regional Product Identity Designation

**DOI:** 10.3390/molecules30122524

**Published:** 2025-06-09

**Authors:** Amner Muñoz-Acevedo, Osnaider J. Castillo, Clara Gutiérrez-Castañeda, Mónica Simanca-Sotelo, Beatriz Álvarez-Badel, Alba Durango-Villadiego, Margarita Arteaga-Márquez, Claudia De Paula, Yenis Pastrana-Puche, Ricardo Andrade-Pizarro, Ilba Burbano-Caicedo, Rubén Godoy

**Affiliations:** 1 Department of Chemistry and Biology, Universidad del Norte, Puerto Colombia 081007, Colombia; osnaiderc@uninorte.edu.co; 2 Department of Microbiology, Universidad Libre, Barranquilla 081007, Colombia; clarag.gutierrezc@unilibre.edu.co (C.G.-C.); ilba.burbano@unilibre.edu.co (I.B.-C.); 3 Department of Food Engineering, Faculty of Agricultural Science, Universidad de Córdoba, Montería 230002, Colombia; msimanca@correo.unicordoba.edu.co (M.S.-S.); bealvarez@correo.unicordoba.edu.co (B.Á.-B.); albadurango@correo.unicordoba.edu.co (A.D.-V.); mrarteaga@correo.unicordoba.edu.co (M.A.-M.); cdepaula@correo.unicordoba.edu.co (C.D.P.); yipastrana@correo.unicordoba.edu.co (Y.P.-P.); rdandrade@correo.unicordoba.edu.co (R.A.-P.); rudago@correo.unicordoba.edu.co (R.G.)

**Keywords:** artisanal dairy food, suero costeño, volatile fractions, FAME profiles, HS-SPME/SDE, PCA/LDA

## Abstract

A traditional dairy product from northern Colombia is suero costeño (SC), typically handmade through artisanal processes involving the natural fermentation of raw cow’s milk (RM); it is characterized by a creamy texture and a distinctive sensory profile, with a sour/salty taste and rancid odor. This study aimed to determine the chemical identity (using GC-FID/MSD) of SC and RM samples (from eight locations in the department of Córdoba-Colombia) by analyzing volatile components (trapped by HS-SPME and SDE) and fatty acid content. Consequently, the most notable results were as follows: (a) myristic (7–12%), stearic (12–17%), oleic (13–23%), and palmitic (21–29%) acids were the most abundant constituents [without significant differences among them (*p* > 0.05)] in both RM and SC fats; these were also expressed as polyunsaturated (2–5%), monounsaturated (26–36%), saturated (59–69%), omega-9 (19–30%), omega-6 (0.5–1.6%), and omega-3 (0.2–1.2%) fatty acids; (b) differences in the composition (*p* < 0.05) of the volatile fractions were distinguished between RM and SC samples; likewise, the SC samples differed (from each other) in their volatile composition due to the preparation processes applied (processes with raw milk and natural fermentation had less variability); nonetheless, it was possible to determine the volatilome for the artisanal product; and (c) the major components responsible for the chemical identity of SC were ethyl esters (of linear saturated and unsaturated acids, short/medium chains), aliphatic alcohols (linear/branched, short/long chains), aliphatic aldehydes (long chains, >C_14_), alkyl methyl ketones (long chains, >C_11_), sesquiterpenes (caryophyllane/humulane types), monoterpenes (mono/bi-cyclics), short-chain fatty acids, and aromatic alcohol/acid, among others.

## 1. Introduction

“Suero costeño (SC)”, with a certain similarity in flavor and texture to sour cream, is a typical food (gastronomic heritage) of the northern region of Colombia and, as a dairy product, it is the result of the aerobic fermentation (spontaneous acidification by native microorganisms) of raw cow’s milk; its preparation method is mainly handmade by peasants in rural areas [1,2,3]. As food, some values of the proximate analysis for SC are 4.0–7.8% (protein), 65–76% (moisture), acidity (0.2–0.5%), 0.3–0.9% (crude fiber), and 3.6–5.8 (pH) [3] and its primary physical and organoleptic characteristics are that it is a colloidal fluid (gel) with little or no syneresis (no stabilizer), a white color, moderate to high viscosity, lumpy (high content of suspended solids—protein and fat), smooth texture, sour/salty taste, and acrid or rancid odor [3,4]. In Colombia, the main producing departments are Córdoba, Sucre, Cesar, Bolivar, and Magdalena [2,3,5].

Regarding the organoleptic characteristics of dairy products (related to consumer acceptance), some of the most important attributes are taste and aroma (flavor), along with mouthfeel, all of which originate through a combination of chemical components [6,7,8]. In this sense, the mouthfeel could be attributed to the fatty acid (FA) and protein contents (related to viscosity and texture) of the food, while the taste to sugars, salts, and short-chain fatty acids (SCFAs), as well as other fermentation by-products. Additionally, aromas are the result of a proper balance of volatile organic constituents, which are produced by thermal degradation, lipid oxidation, and fermentation by lactic acid bacteria (LAB) [6,9,10,11,12,13]. These odorant molecules are usually hydrophobic (some polar), with molar weights up to 300 g/mol, and become impact compounds because they impart distinctive notes to foods (e.g., esters: fruity; C_4_–C_10_ FAs and aldehydes: rancid; terpenoids: musty, earthy, and herbaceous; aromatic alcohols, aldehydes, and acids: floral; lactones: coconut-like) [9,14]. Thus, flavor is, among others, the most relevant attribute for the acceptance of SC and depends on both the fermentation and production processes, as well as on the quality and type of raw material (e.g., milk) [2,9,14].

As mentioned above, SC has a texture and taste like sour cream, which is a heavy, viscous, shiny product with a balanced, pleasant, butter-like aroma, as reported by Meunier-Goddik [15] and Shepard et al. [16]. The characteristic odor of the sour cream is due to diacetyl, acetoin, acetic and butyric acids, acetaldehyde, and/or dimethyl sulfide, but it may also contain aldehydes, ketones, alcohols, fatty acids, lactones, esters, nitrogen-containing compounds, and aromatic hydrocarbons contributed by the raw milk [17,18]. When reviewing the scientific literature on FA profiles and aroma-related volatile compounds for SC, only one record was found indirectly related to this topic (Valencia Garcia et al. [19]); however, there were other reports for SC focused on the microbiological diversity (selection, identification, and isolation), characterization, and biological effect of LAB and yeasts, starter cultures, spontaneous fermentation, physicochemical and rheological characterizations, etc. [3,4,5,20,21,22,23,24,25,26,27,28,29]. Based on these works, 22 strains of bacteria and yeasts have been isolated, e.g., *Enterococcus faecium*, *Lactobacillus acidophilus*, *L. brevis*, *L. delbrueckii*, *L. lactis*, *L. paracasei*, *L. pentosus*, *L. plantarum*, *Leuconostoc lactis*, *Streptococcus infantarius*, etc., which would be responsible for some of the physicochemical and organoleptic characteristics of SC.

Finally, according to the European Commission-Agriculture and Rural Development, when a product (food, wine, or agricultural products) is traditionally and entirely manufactured (preparation, processing, and production) in a specific region and acquires unique properties, it can be registered for a protected designation of origin (PDO). Nonetheless, this product must have unique properties (physicochemical and organoleptic, among others) identified and established, e.g., odor, taste, color, texture, viscosity, chemical constituents, etc., in addition to meeting the above criteria [30]. Colombia, through its Ministry of Industry and Commerce, has conferred seals for a denomination of origin that protects unique Colombian products with an established identity (foods (cheese and fruits), flowers, handicrafts, etc.) [31]. As a first approach to establish the identity of SC as a regional autochthonous product, this work determined the chemical composition by GC-FID/MSD of volatile fractions (by HS-SPME and SDE) and the fatty acid content (by chemical derivatization to FAMEs)—as a chemical fingerprint—of both suero costeño and raw milk from eight localities in the department of Córdoba (Colombia), where farmers produced SC from cow’s milk and five manufacturing processes, which could influence the production of SC volatiles. In addition, this study made it possible to relate the effect of the SC manufacturing process and raw material on the development of aroma-related volatile constituents.

## 2. Results

### 2.1. Fat Content and Fatty Acid Profiles

**Figure 1:**
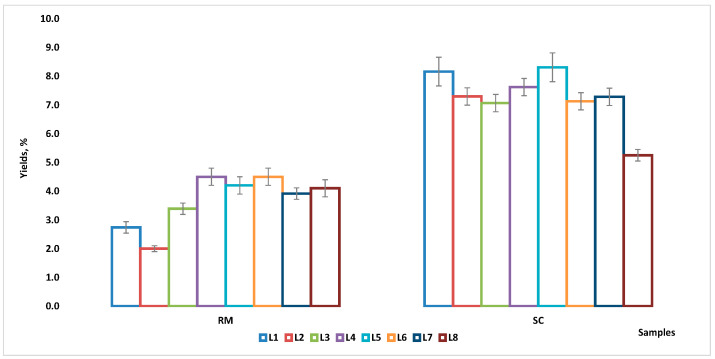
Yields (%) of fat content for the RM and SC samples (measure unit: mean ± standard deviation).

After isolation of RM and SC fats by the Rose–Gottlieb method, their yields were determined (Figure 1). Thus, 2.0 ± 0.1–4.5 ± 0.3% and 5.2 ± 0.2–8.3 ± 0.5% were the fat contents for RM and SC, respectively, and the average values for each type of samples were 3.7 ± 0.9% (RM) and 7.3 ± 0.9% (SC). Moreover, there were higher variations in fat content among the milk samples, but in the SC samples the variations were smaller; the greatest variation was found in the sample from location 8 (SC prepared from raw milk spontaneously fermented for one day in “totuma”) with the lowest yield (5.2 ± 0.2%). Nonetheless, the ratios between SC and RM yields indicated that the fat content in the SC samples was higher in most cases (1.3 to 3.6 times).

The result of the Shapiro–Wilks (S-W) test for the raw data showed that both RM and SC yields had a normal distribution (*p*: 0.1714; *p*: 0.1503—Table S1), while the results of the non-parametric ANOVA showed that there were significant differences (*p* < 0.0001, Friedman (F) test—Table S2) between the yields of RM compared to those of SC, but within each group (RM and SC) there were no differences (*p* > 0.999, Kruskal–Wallis (K-W) test—Table S3).

**Figure 2:**
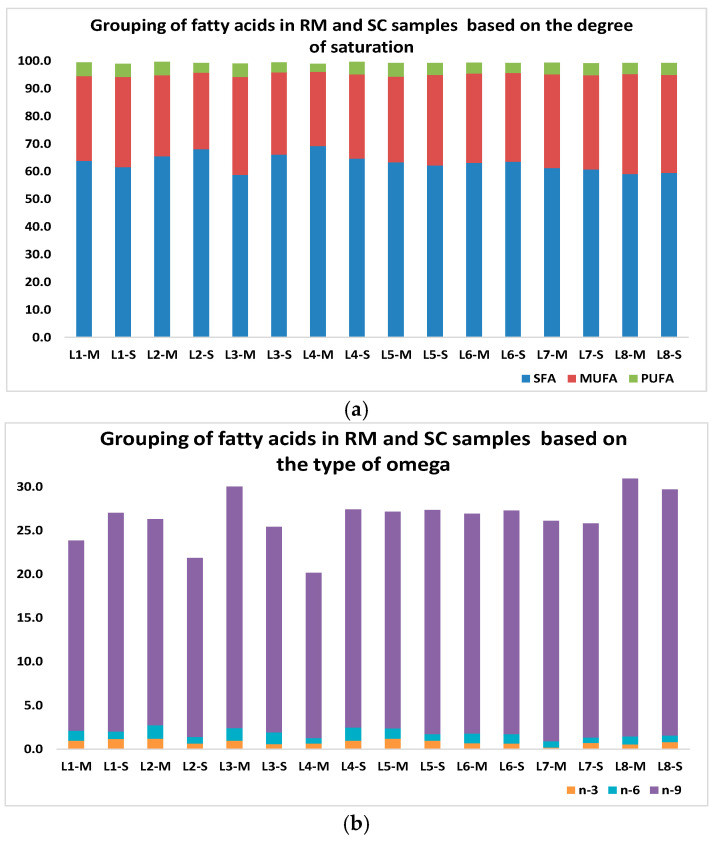
Grouping of FAs in RM and SC samples based on (**a**) degree of saturation and (**b**) type of omega. SFAs: saturated fatty acids, MUFAs: monounsaturated fatty acids, and PUFAs: polyunsaturated fatty acids (n-3 acids, n-6 acids, and n-9 acids).

Concerning the fatty acids of the RM- and SC-fat fractions, these were characterized as methyl esters (FAMEs) (Figure S1) and reported (>99%) in Table S4. According to the Table, the FAs of the samples were similar, with some differences in their relative amounts, with C_6_–C_25_ fatty acids (saturated and unsaturated) being representative in all samples; thus, the predominant FAs in the RM and SC samples in order of abundance were palmitic (C_16:0_; 21–29%), oleic (C_18:1_ *cis*-Δ^9^; 13–22%), stearic (C_18:0_; 12–17%), and myristic (C_14:0_; 7–12%) acids (Figure S2) and the total content of saturated (SFAs), monounsaturated (MUFAs), and polyunsaturated (PUFAs) fatty acids ranged between 59–69%, 27–36%, and 2.9–5.0%, respectively (Figure 2a). In addition, the contents of n-3, n-6, and n-9 acids were 0.2–1.2%, 0.6–1.5%, and 19–30%, in that order, for both RM and SC (Figure 2b). Another important fact is that a non-negligible percentage (7–17%) of C_18:1_ octadecenoic acid isomers (*Z*/*E*) was determined in all samples, as well as one n-3 pentaunsaturated fatty acid (EPA isomer) [32] and some branched and odd-numbered fatty acids (C_9:0_–C_25:0_).

On the other hand, the atherogenic (AI) and thrombogenic (TI) indices, which are dietary risk values for cardiovascular disease related to the platelet aggregation inhibition (AI) and blood clot formation (TI) [33], were calculated for each sample. Thus, the AI and TI values were 1.4–2.8 and 2.0–3.3 for both RM and SC. Statistical analysis of these data using S-W, Friedman, and K-W tests (Tables S5–S13) indicated that the four main FAs, along with the classification by FA families and the AI and TI values of both types of samples (RM and SC), had a normal distribution (*p*: 0.3497–0.9850, *p*: 0.1209–0.6500, and *p*: 0.3186–0.5202); and there were no significant differences within and between all samples (Friedman: *p*: 0.9685 and 0.8546—major FA, *p*: 0.3251 and 0.4986—families of FAs; KW: *p*: > 0.9999—major FA, *p*: 0.4514—families of FAs, *p*: 0.4514—AI and TI values). However, there were significant differences for AI and TI values within the SC samples (*p* < 0.0001—Friedman test) but not for the RM samples (*p*: 0.0672—Friedman test).

### 2.2. Chemical Composition of Volatile Fractions

The chemical constituents positively identified (83–99%) in the volatile fractions of SC and RM samples by SDE/SPME-GC-FID/MSD (Figures S3 and S4) are listed in order of elution in Table A1 and Table A2. Thus, the volatile profiles of the RM and SC samples were, respectively, composed of 48–78 and 65–100 constituents. The common major constituents (1–46% of the relative amounts by SDE—Table A1) found in the volatile fractions of milk samples were nine fatty acids (C_8:0_–C_16:0_, including C_10:1_ and C_14:1_) together with benzoic acid. However, some minority non-acid compounds, related to the organoleptic properties of RM, were also recognized, e.g., six methyl alkyl ketones, five cyclic esters (lactones), and four aliphatic aldehydes. In addition, some miscellaneous compounds such as two sesquiterpenes (β-caryophyllene, α-humulene), 10 linear/branched hydrocarbons, one aromatic hydrocarbon, and one aromatic alcohol (benzenethanol) were present. Non-parametric ANOVA of these data indicated that there were no significant differences within each group [type of compounds (*p*: 0.4544, K-W test—Table S14)] for all RM samples, but there were differences (*p*: <0.05, Friedman test—Table S15) in the chemical composition of sample L1 compared to the other milk samples (no significant differences between them, *p*: > 0.05).

Furthermore, the volatile fractions by HS-SPME of the RM samples (Table A2) were mainly represented by C_10:0_, C_8:0_, C_6:0_, and C_12:0_ fatty acids together with limonene, the C_2:0_ and C_4:0_ acids, isoamyl alcohol, 2-nonanone, benzoic acid, the C_14:0_ and C_14:1_ fatty acids, acetone, and pentanol. Other types of compounds related to milk flavor were also present: five methyl alkyl ketones, four cyclic esters, two sesquiterpenes, seven linear/branched hydrocarbons, benzenethanol, and myristylaldehyde. It is important to mention that ester-type compounds (ethyl caprate, ethyl laurate, and ethyl myristate) were identified in the milk samples, as well as monoaromatic hydrocarbons (toluene and styrene), and a high content of 2-nonanone in milk sample L7. As with the SDE method, the non-parametric ANOVA of HS-SPME data showed the same trend; i.e., there were differences (*p*: < 0.05, Friedman test—Table S15) for the composition of sample L1 versus the remaining milk samples (no significant differences between them, *p*: > 0.05), but there were no differences within each group [type of compounds (*p*: 0.4544, K-W test—Table S14)] for all samples. Non-parametric ANOVA applied to the compositions of volatile fractions (average relative amounts per compound family for all RM samples) for both SDE and HS-SPME methods revealed no significant differences within and between all samples (Friedman: *p*: 0.6507; K-W: *p*: 0.4544—Tables S16 and S17).

Considering the chemical compositions of the volatile fractions by SDE (Figure S4A) of the SC samples, these were mainly typified by 13 fatty acids (including short-chain) [(3-Me)-C_4:0_ (iso-C_5:0_), (2-Me)-C_4:0_, C_5:0_, C_6:0_-C_16:0_, C_10:1_, and C_14:1_], six ethyl esters (e.g., ethyl caprate, ethyl 9-decenoate, etc.), three aliphatic alcohols (e.g., (2*E*)-tridecen-1-ol, etc.), six methyl alkyl ketones, four aliphatic aldehydes, five sesquiterpenes (e.g., selinane, cadinene isomers, etc.), eight linear/branched hydrocarbons, five lactones, one aromatic acid, and one aromatic alcohol. Other relevant molecules (volatiles) were also identified in the majority (50–75%) of the SC samples, i.e., 11 esters (e.g., ethyl caprylate, ethyl (9*Z*)-tetradecenoate, ethyl caproate, β-phenethyl acetate, ethyl (9*Z*)-heptadecenoate, etc.), two aromatic hydrocarbons, three aliphatic aldehydes, four branched/linear hydrocarbons, and three monoterpenes (camphene, fenchene, and α-pinene), along with C_16:1_ fatty acid, *m*-tolualdehyde, and octadecanol; therefore, the total constituents identified in the SC samples by SDE were 53–75 compounds. Nonetheless, if the compositions (by SDE) of SC samples are contrasted with those of the RM, and the common components are subtracted (so that those molecules that appeared after the fermentation process stand out, some of which are necessarily the result of the fermentation biochemistry (by LAB) but others are not), the new contributing composition related to the aroma profile could involve between 17–30 components, without disregarding the contribution of the inherent components of the RM. Non-parametric ANOVA of SDE data from SC samples revealed significant differences (*p*: < 0.05, Friedman test—Table S18) for the compositions of samples L3 vs. L6 (related to ester content) and some differences between L2 vs. L4 (related to ester and fatty acid contents); and, based on the results of the K-W test, there were no differences within each group [type of compounds (*p*: 0.4557—Table S19)] for all samples.

On the other hand, the volatile fractions isolated by HS-SPME (Figure S4B) from the SC samples were mostly represented by 11 fatty acids (including short-chain; C_2:0_, C_4:0_, C_6:0_–C_16:0_, C_10:1_, and C_14:1_), seven ethyl esters (e.g., ethyl caprate, ethyl caprylate, ethyl acetate, ethyl laurate, ethyl caproate, etc.), one aromatic acid, five sesquiterpenes, four methyl alkyl ketones, one aromatic alcohol, one monoterpene (limonene), one methyl alkoxyl ketone (acetoin), four aliphatic alcohols (e.g., ethanol, isoamyl alcohol, (2*E*)-tridecen-1-ol, etc.), seven linear/branched hydrocarbons, three lactones, and two aliphatic aldehydes (myristylaldehyde and palmitaldehyde). Lastly, a sesquiterpene [unidentified (C_15_H_16_—M^+•^ 206, base peak 191), retention index: 1470, and non-negligible amount relative (0.6–12% by SPME; 0.4–5% by SDE)] was present in all SC sample.

The other volatile molecules identified and related to 50–75% of SC samples were 10 esters (e.g., ethyl 9-decenoate, ethyl butyrate, ethyl pelargonate, ethyl tridecanoate, ethyl (9*Z*)-hexadecenoate, isopropyl palmitate, etc.), four aliphatic alcohols (e.g., (2*E*)-undecen-1-ol, pentanol, etc.), two methyl alkyl ketones, four monoterpenes (α-pinene, *p*-cymene, tricyclene, and camphene), four aliphatic aldehydes, five linear/branched hydrocarbons (e.g., (7*Z*)-tetradecene, neophytadiene, etc.), three fatty acids (e.g., (5*Z*)-C_12:1_, C_11:0_, etc.), two sesquiterpenes (cyperene and *trans*-calamenene), and one lactone (δ-tetradecalactone), along with *m*-tolualdehyde and styrene. Consequently, the most frequent constituents of SC samples by HS-SPME were 48–79 compounds, but the subtraction of the common components between RM and SC would indicate that 15–42 constituents would contribute to the flavor-related composition of the SC samples, together with those corresponding to the RM. The results of non-parametric ANOVA on the composition data of SC samples showed that there were no significant differences within each group [type of compounds (*p*: 0.4579—Table S19)] for all samples based on the K-W test, while according to the Friedman test (Table S18), significant differences (*p*: 0.0075) were observed between samples L3 and L6, L1 and L6, and L8 and L3, as well as some differences between the remaining samples.

### 2.3. Differences and Similarities Between Constituents of Volatile Fractions by PCA and LDA

**Figure 3:**
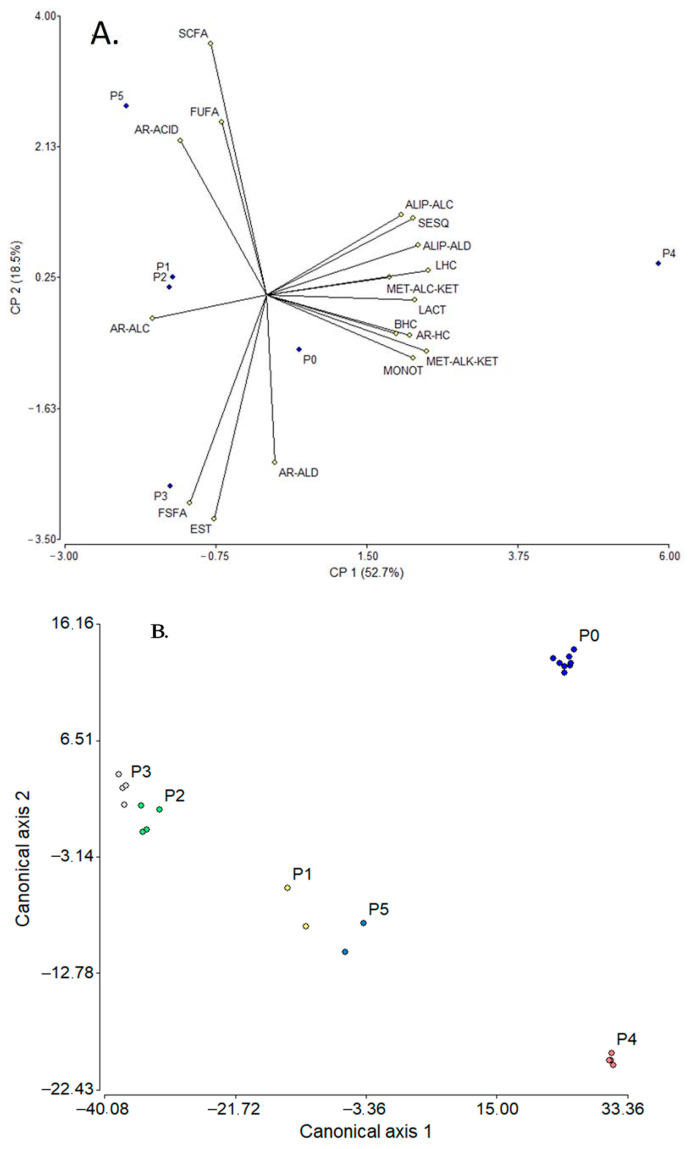
PCA (**A**) and LDA (**B**) plots relating the effect of SC manufacturing method to the composition of volatile fractions. SCFAs: short-chain fatty acids (≤C_6_), FSFAs: free saturated fatty acids (≥C_7_), FUFAs: free unsaturated fatty acids, ALIP-ALDs: aliphatic aldehydes, ALIP-ALCs: aliphatic alcohols, MET-ALK-KETs: methyl alkyl ketones, AR-ALDs: aromatic aldehydes, AR-ALCs: aromatic alcohols, AR-ACIDs: aromatic acids, ESTs: esters, LACTs: lactones, MONOTs: monoterpenoids, SESQs: sesquiterpenes, LHCs: linear hydrocarbons, BHCs: branched hydrocarbons, AR-HCs: aromatic hydrocarbons, HTC-ALCs: heterocyclic alcohols, PhOHs: phenols, HTC-ETs: heterocyclic ethers, MET-ALC-KETs: methyl alcohol ketones, AL-ACIDs: aliphatic acids, P: process related to the method for obtaining suero costeño; P0: unfermented raw milk, and P1, P2, P3, P4, and P5: processes described in Table S20.

An important aspect in these results was the difference or similarity found between the SC samples (regardless of extraction method), which could be mainly attributed to the manufacturing process (including raw material and fermentation time and mode, as well as the inoculum type) of this food product (Table S20). Based on this Table, there were at least five SC preparation methods. Consequently, it was hypothesized that the chemical compositions should be similar for L2 and L8, L3 and L7, L4 and L6, with some difference for L5. For L1, it could show similarity with L2 and L8, but its fermentation was longer (for 72 h). Therefore, the comparison between the volatile fractions of the RM samples with those of SC was carried out by multivariate analysis using PCA and discriminant analysis based on the type of compounds (constituents of the volatiles fractions) and the manufacturing process of the SC. The resulting plots from the PCA and LDA are shown in Figure 3.

Thus, the PCA explained ca. 71% of the data variability as a function of the two main factors; the variables with the highest contribution to PC1 were linear hydrocarbons (LHCs—0.98), methyl alkyl ketones (MET-ALK-KETs—0.97), aliphatic aldehydes (ALIP-ALDs—0.92), and lactones (LACTs—0.90), while for PC2, the contributing variables were short-chain fatty acids (SCFAs—0.87), esters (EST—0.77), and free saturated fatty acids (FSFAs—0.70). These correlations allowed to discriminate each process used for SC manufacture; i.e., P4 [cooked whey residue from cheese and salting whey (or commercial milk cream)] was the process with the greatest difference (according to the chemical composition criterion), clearly observed in the graph (Figure 3A), while the most similar processes were P1 (milk fat and spontaneous fermentation, three day) and P2 [raw milk and spontaneous fermentation, one day (“totuma”)], closely grouped. It is important to mention that for this analysis the “point 0” (P0, related to unfermented RM) was included, which was used for comparison. On the other hand, Figure 3B displays the graph resulting from the LDA applied to all samples. According to this analysis, ca. 98% of the data variability could be explained considering the two canonical axes 1 (83.8%) and 2 (14.2%); the most discriminating variables were FSFAs (75.6) and ESTs (−79.0) for axis 1 and monoterpenes (MONOTs, 7.5) and methyl alkoxyl ketones (MET-ALC-KETs, −6.7) for axis 2. Additionally, it was evident that P0 differed noticeably from the other processes and three groupings could be observed: (i) P2 and P3, with greater similarity; (ii) P1 and P5, some similarity; and (iii) P4, the most dissimilar.

**Figure 4:**
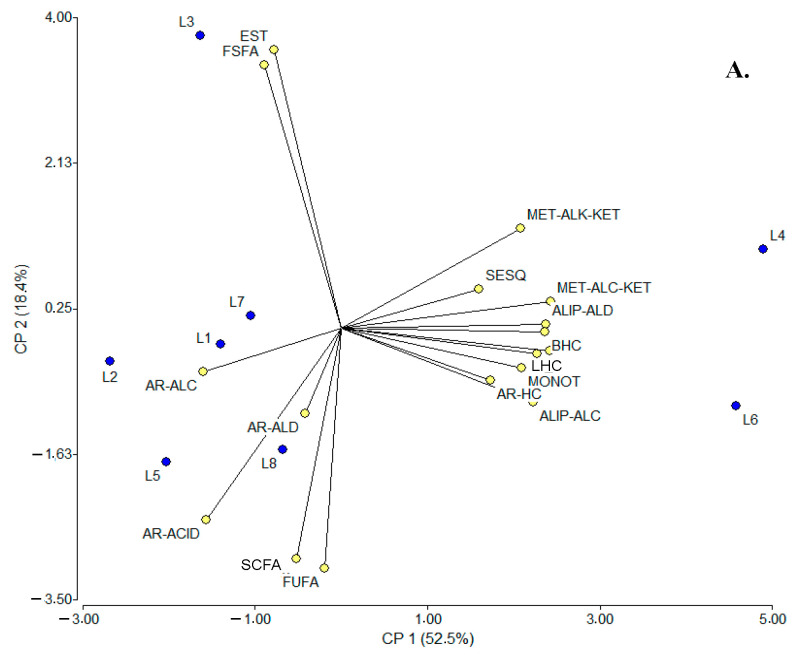

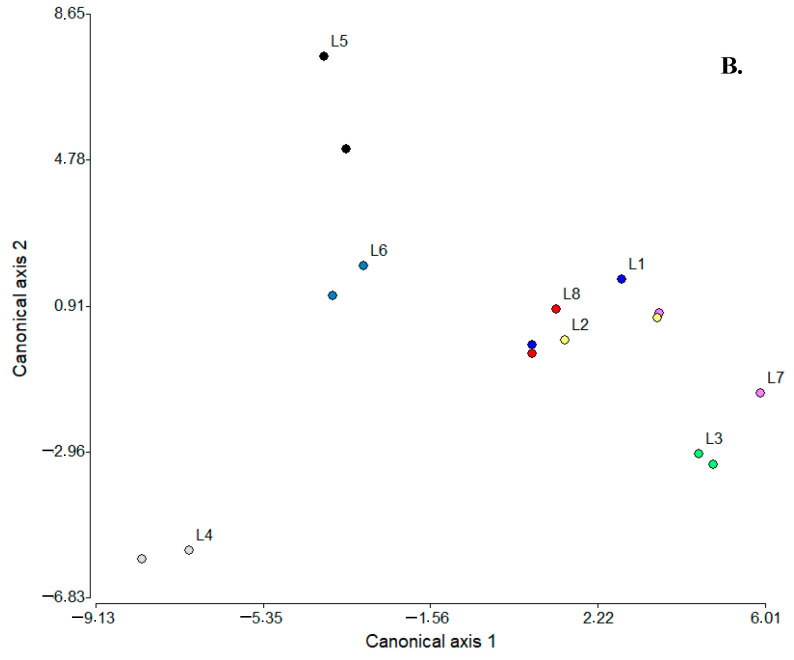
PCA (**A**) and LDA (**B**) plots relating SC sampling points (locations) to the composition of volatile fractions. SCFAs: short-chain fatty acids (≤C_6_), FSFAs: free saturated fatty acids (≥C_7_), FUFAs: free unsaturated fatty acids, ALIP-ALDs: aliphatic aldehydes, ALIP-ALCs: aliphatic alcohols, MET-ALK-KETs: methyl alkyl ketones, AR-ALDs: aromatic aldehydes, AR-ALCs: aromatic alcohols, AR-ACIDs: aromatic acids, ESTs: esters, LACTs: lactones, MONOTs: monoterpenoids, SESQs: sesquiterpenes, LHCs: linear hydrocarbons, BHCs: branched hydrocarbons, AR-HCs: aromatic hydrocarbons, HTC-ALCs: heterocyclic alcohols, PhOHs: phenols, HTC-ETs: heterocyclic ethers, MET-ALC-KETs: methyl alcohol ketones, AL-ACIDs: aliphatic acids, and DIKETs: diketones. L: locations where suero costeño samples were collected; L1–L8: locations listed in Table S20.

From another perspective, an additional multivariate analysis was performed using the same statistical tools to contrast the volatile fractions between SC samples and locations. For PCA (Figure 4A), the main findings were that the two main factors could explain ca. 71% of the data variability; the contributing variables to PC1 were LHCs and MET-ALC-KETs (0.98 for each) and branched hydrocarbons (BHCs) and ALIP-ALDs (0.96 for each), while for PC2, these were ESTs (0.86), FSFAs (0.82), and free unsaturated fatty acids [FUFAs (−0.74)]. As a result of this analysis, three groupings could be established: (i) L4 and L6, (ii) L3, and (iii) L1, L2, L5, L7, and L8; among them, L4 and L6, L2 and L8, and L1 were consistent with the hypothesis of compositional similarity based on the same manufacturing process, but L5, L7, and L3 apparently were not. Considering the LDA, the two canonical axes 1 (58.0%) and 2 (36.0%) were able to explain ca. 94% of the data variability; the most discriminating variables for both axes (1 and 2) were FSFAs (−4.70 and 7.21) and ESTs (4.42 and −7.17). In addition, four groupings were observed in the plot: (i) L3 and L7, closely related to (ii) L1, L2, and L8, (iii) L5 and L6, and (iv) L4. Considering the compositional similarity hypothesis, then, L3 and L7 and L2, L8, and L1 were consistent, but L4, L5, and L6 were not.

Finally, the results of the “two-sample inference (F-test of equality of variances)” for all data, grouped by matrix types (RM and SC), are listed in Table S21. According to the table, seven types of compounds showed significant differences (*p* <0.05) between the samples and were, therefore, the differentiating and outstanding component types: free saturated fatty acids, esters, aliphatic aldehydes, methyl alkoxyl ketones, aromatic aldehydes and acids, and branched hydrocarbons.

**Figure 5:**
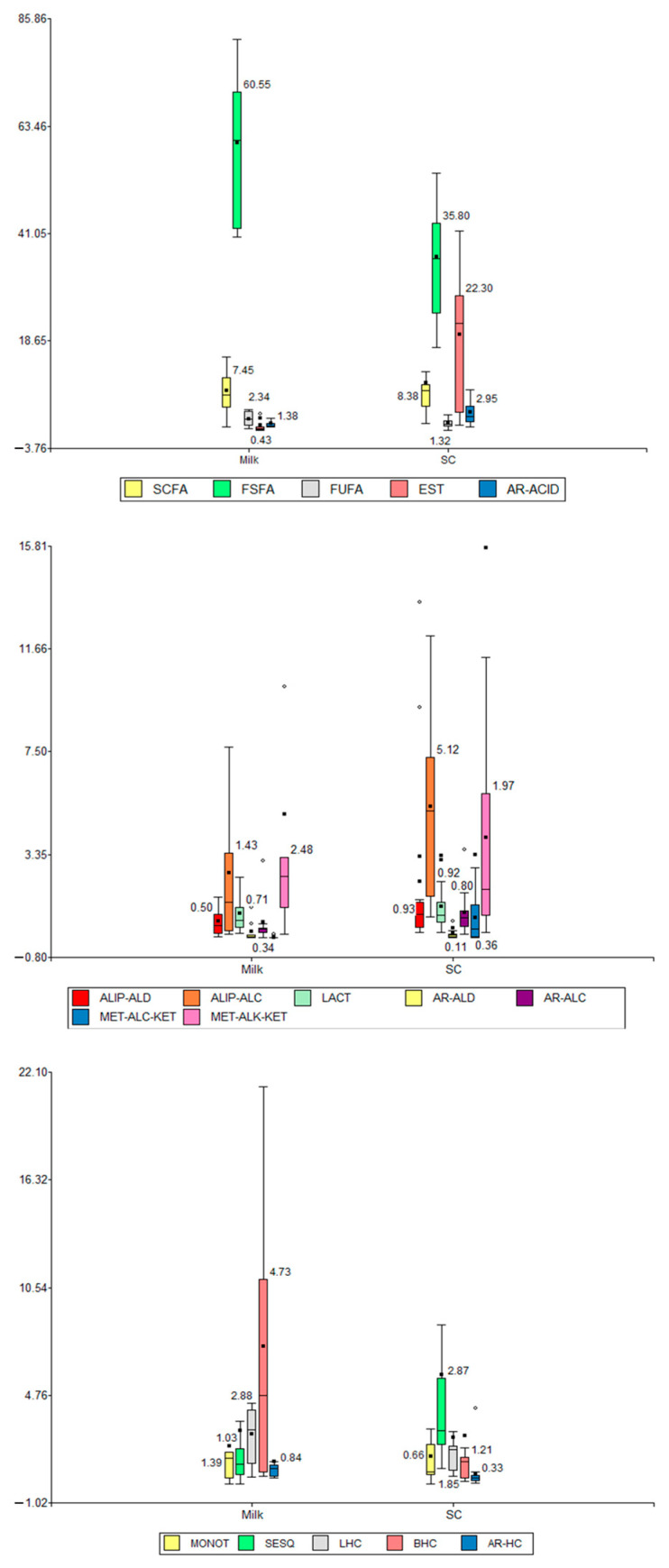
Box plots relating compound types (volatile fractions) versus matrix types (milk and SC). SCFAs: short-chain fatty acids (≤C_6_), FSFAs: free saturated fatty acids (≥C_7_), FUFAs: free unsaturated fatty acids, ESTs: esters, AR-ACIDs: aromatic acids, ALIP-ALDs: aliphatic aldehydes, ALIP-ALCs: aliphatic alcohols, LACTs: lactones, AR-ALDs: aromatic aldehydes, AR-ALCs: aromatic alcohols, MET-ALC-KETs: methyl alcohol ketones, MET-ALK-KETs: methyl alkyl ketones, MONOTs: monoterpenoids, SESQs: sesquiterpenes, LHCs: linear hydrocarbons, BHCs: branched hydrocarbons, and AR-HCs: aromatic hydrocarbons.

Box plots were also applied to all data, comparing matrix types versus compound types (Figure 5). From these plots, it could be observed and inferred that the content (i) distinctly decreased for SFSAs, FUFAs, and BHCs, (ii) notably increased for ESTs, ALIP-ALCs, MET-ALK-KETs, MET-ALC-KETs, and SESQs, (iii) slightly decreased for SCFAs, LHCs, and AR-HCs, (iv) increased slightly for AR-ACIDs, ALIP-ALDs, AR-ALCs, AR-ALDs, and MONOTs, and (v) did not change for LACTs in SC samples compared to milk samples. This interpretation is important because it showed the formation of ESTs from SFSAs, FUFAs, and SCFAs and ALIP-ALCs; i.e., the products (esters) increased and the precursors/reagents (acids and alcohols) decreased.

## 3. Discussion

As a starting point for this discussion, when comparing the results of the yields and fat contents determined for the RM samples with the available scientific literature, some similarities and differences were found. For example, Eisenstecken et al. [34], Mehta [35], Nantapo et al. [36], O’Callaghan et al. [37], and Villeneuve et al. [38] affirmed that cow’s milk contained fat in the range of 2.3–5.3%, which was consistent with the fat content of most of the RM samples in this work, except for that at location 2 (2%), which was outside but close to the lower limit of yields. Furthermore, the FA profiles of raw cow’s milk from the literature reviewed were similar to those of this study; i.e., the main fatty acids reported by Eisenstecken et al., Mehta, Villeneuve et al., Čuboň et al. [39], Liu et al. [40], MacGibbon and Haddadian [41], Pilarczyk et al. [42], Riuzzi et al. [43], and Santos Júnior et al. [44], were palmitic (19–39%), oleic (12–34%), stearic (6–15%), and myristic (7–14%) acids.

Likewise, the contents of SFAs, MUFAs, PUFAs, and omega acids (n-3 and n-6) in this research were mostly within the ranges reported by Nantapo et al. [36], Pilarczyk et al. [42], and Butler et al. [45], which were SFAs (48–78%), MUFAs (2–30%), PUFAs (2–9%), n-3 (0.3–2%), and n-6 (1–3%); nonetheless, the values of MUFAs and n-3 and n-6 in this study were respectively higher (~27–36%) and lower (0.2–1.2% and 0.6–1.5%) than those recorded by Mollica et al. [46] (MUFAs: 2–30%, n-3: 0.3–1.8%, and n-6: 1.2–3.0%). In addition, Santos Júnior et al. [44] found values of n-3 and n-6 for milk samples (raw/pasteurized) of 2.2–3.2% and 0.4–0.5%, in that order, showing differences with this work. In contrast, Riuzzi et al. [43] reported n-3 and n-6 values for RM samples of 0.9–1.2% and 2.3–2.8%, correspondingly, which were similar and/or higher than those of this work; furthermore, SFAs (67–70%), MUFAs (26–28%), and PUFAs (4.6–5.2%) values showed some differences in SFA (higher values) and MUFA (lower values) contents.

Finally, the calculated AI and TI values were compared with the indices determined by O’Callaghan et al. and Pilarczyk et al. [37,42]; the values reported by these authors ranged between 2.4–3.6 for AI, and 2.2–4.6 for TI, which were higher than or equal to those of this research. The fundamental premise of these indices is that the lower the AI and TI values, the lower the risk of cardiovascular disease [33].

Since SC is an artisanal product from Colombia, only one report has been found (Valencia García et al. [19]) that mentions the chemical analysis of the FA profiles of SC samples manufactured at laboratory and producer scales using three different procedures; nonetheless, no chemical composition related to the FAs was included in that manuscript. Consequently, this would be the first report on yields and fatty acid composition for SC. For this reason, these contents or compositions will be compared and discussed with other artisanal products (like SC) from other countries and with commercial sour creams or fermented creams.

Thus, Yilmaz-Ersan [47] determined the FA profiles and families of commercial UHT creams fermented with three probiotic strains (*Bifidobacterium lactis*, *L. acidophilus*, and *L. rhamnosus*); the creams were composed of SFAs (64–65%), MUFAs (28%), and PUFAs (4%), represented by C_16:0_ (30–31%), C_18:1_ (22–23%), C_18:0_ (12%), and C_14:0_ (10%) acids. Moreover, Izsó et al. [48] reported the yield (20%) of three types of sour cream, as well as their FA contents (70%-SFAs, 23%-MUFAs, 3%-PUFAs, 0.4%-n-3, and 2.2%-n-6; characterized by C_16:0_-33%, C_18:1_-20%, C_14:0_-12%, and C_18:0_-10%). Comparisons between SC and sour creams, related to fatty acid contents and compositions, presented similarities but differed in fat yields; that is, sour creams had fat contents of 18–20% (this is a quality/manufacturing requirement ranging from 14–35% [16,49]) while SC had lower fat content values (5–8%).

**Figure 6:**
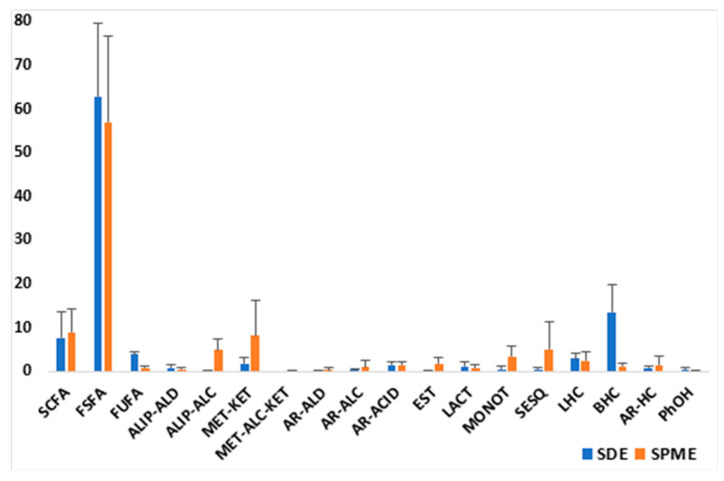
Comparison of the types of compounds identified in the volatile fractions (by SDE and HS-SPME) of the RM samples (average values of the relative amounts).

In the aspects related to raw milk volatiles, the comparison between the compounds obtained by SDE and HS-SPME showed some similarities and differences both in relative amounts and in certain constituents, indicating that both techniques were complementary (Figure 6). That is, some components were trapped by SPME but not by SDE, e.g., limonene, acetone, isoamyl alcohol, pentanol, ethyl caprate/laurate/myristate, and acetic and butyric acids; and other components were obtained by SDE but not by SPME, e.g., γ-dodecalactone, octadecanal, palmitaldehyde, linear/branched/aromatic hydrocarbons, etc. Nevertheless, the most abundant constituents for both methods were similar, with differences in their relative amounts.

The gamma of volatile constituents found in the volatile fractions (by SDE and HS-SPME) of the milk samples was consistent with the scientific literature consulted, with some differences. Thus, Yuan et al. [50] reported that Chinese raw milk mainly contained fatty acids (C_15:0_, C_16:0_, C_14:0_, C_10:0_, and C_6:0_), followed by styrene, benzoic and butyric acids, and 2-nonanone, while another milk sample studied by Zhang et al. [51] was constituted by C_8:0_, C_6:0_, C_10:0_, C_2:0_, and C_4:0_ fatty acids together with acetoin and other ketone and aldehyde compounds (low molecular weight—LMW), together with δ-decalactone/δ-dodecalactone, as the main volatile components.

Instead, in some Italian milk samples, Zacometti et al. [52] identified fatty acids (C_10:0_, C_8:0_, C_7:0_, and C_3:0_), LMW-aldehydes, 2-octanone, anisyl formate, and 2-pentanol as volatile constituents. Similarly, in another study by Genovese et al. [53], the authors reported that the volatile fractions of RM samples consisted of C_8:0_, C_6:0_, C_10:0_, and C_4:0_ fatty acids, along with hexanal, 2-ethylhexanol, nonanal, acetone, and 2-butanone.

In contrast, Vagenas and Roussis [54] indicated that the volatile fraction of Greek RM (by HS-SPME) was composed of fatty acids (C_8:0_, C_6:0_, C_10:0_, C_4:0_, and C_12:0_) as major constituents and methyl alkyl ketones and lactones as minor components. Additionally, Villeneuve et al. [38] determined that Canadian RM volatiles were represented by fatty acids (C_10:0_, C_6:0_, C_8:0_, C_12:0_, and C_2:0_), followed by methyl alkyl ketones (acetone, 2-butanone, 2-heptanone, etc.), aliphatic and aromatic aldehydes, aliphatic esters (methyl- and ethyl-), lactones (δ- and γ-), terpenes (α-pinene and limonene), and alcohols. Finally, Kim Ha and Lindsay [55] determined that RM fat had C_4:0_, C_10:0_, C_6:0_, and C_8:0_ fatty acids as major volatiles.

From a general point of view on volatiles in the SC samples, some particular and unusual molecules (both in high and low relative amounts) were identified, e.g., (7*Z*)-tetradecene, (2*E*)-tridecen-1-ol, ethyl esters [of 9-C_10:1_, (9*Z*)-C_14:1_, (9*Z*)-C_16:1_, (9*Z*)-C_17:1_, (9*Z*)-C_18:1_, (9*Z*,12*Z*,15*Z*)-C_18:3_, (9*Z*,12*Z*)-C_18:2_, and C_18:0_], β-phenethyl acetate, *m*-tolualdehyde, 5-methyl-2-hexanone, 2-methylbutanoic acid, etc. Some of them [(7*Z*)-tetradecene, (2*E*)-tridecen-1-ol, ethyl 9-decenoate (C_10:1_), and ethyl (9*Z*)-tetradecenoate (C_14:1_)] are recorded for the first time as constituents of a dairy product (or fermented sour creams), but others have been found in other dairy products (goat cheese: ethyl 9-decenoate; goat cheese/mozzarella/camembert: β-phenethyl acetate; Spanish artisanal cheese: ethyl (9*Z*)-hexadecenoate, ethyl (9*Z*)-tetradecenoate, ethyl linoleate, and ethyl oleate) and in plants, which could be food sources for animals during grazing; likewise, sesquiterpenes (β-caryophyllene, α-humulene, etc.) and monoterpenes (limonene, α-pinene, *p*-cymene, etc.) found in the SC samples could come from this type of source [56,57,58,59,60].

When consulting the scientific literature on the volatiles of SC, only the report by Valencia García et al. [19] described the volatile profiles (by HS-SPME/GC-MS) of seven SC samples prepared at laboratory (four samples) and production (three samples) scales from isolated native starter cultures (*L. plantarum*, *S. infantarius*, and *L. lactis*). However, no report was found on the chemical composition of the volatile fraction of SC by SDE/GC-MS; therefore, this is the first report on the complete volatilome of this Colombian artisanal food product considering that the SPME technique alone is not sufficient to determine the volatile constituents of the SC, as mentioned above. Comparison between the volatiles identified in SC samples by Valencia García et al. [19] with those of this study showed differences and some similarities. That is, the authors reported for each sample 1–2 alcohols (hexanol, 1,4-butanediol), 1–2 methyl alkoxyl ketones (acetoin, acetol), 1–2 SCFAs (C_2:0_, C_4:0_), 1 FA (C_10:0_), 2–5 aldehydes (acetaldehyde, nonanal, etc.), and 2–3 other components (dimethylsulfide, etc.), for a total of 18 compounds for all samples, with varying relative amounts (0.01–18%); of these volatiles, only acetoin, C_2:0_, C_4:0_, C_10:0_, and nonanal were identified in this research. Furthermore, esters (including lactones), ethanol, fatty (iso-C_4_–C_9_ and C_10:1_–C_16_) and aromatic acids, methyl alkyl ketones, terpenes (C_10_ and C_15_), and hydrocarbons were not identified in any of the SC samples in the study by Valencia García et al. [19]; these notable compositional differences could be related to both the inoculation (strains and time) and the manufacturing processes used for SC production.

Additionally, some comparisons were made with science reports of products with similar physicochemical characteristics to those of SC and related to the fermentation process of cow’s milk (sour cream). Thus, Zhao et al. [61] characterized the profiles of volatiles produced during milk fermentation by *Streptococcus thermophilus* and *Lactobacillus helveticus*); the authors reported 56 constituents, belonging mainly to aldehyde, ketone, acid, alcohol, and ester type compounds. Among them, 2,3-butanedione, acetoin, 2,3-pentanedione, C_6:0_, C_2:0_, acetaldehyde, and C_4:0_ were identified as discriminating molecules between different time points and fermented milk samples.

While Tan et al. [62] identified 36 volatile compounds (14 ketones, 11 aldehydes, six alcohols, and five other compounds) in fermented milk, but as the milk fat content increased the level of ketone compounds increased; in addition, 14 constituents (eight ketones and six aldehydes) were responsible for the aroma profile. Additionally, in the research carried out by Wang et al. [63] on the release profile of flavor compounds from sour cream, the authors found that 31 components such as esters (nine), aldehydes (six), acids (three), alcohols (six), ketones (five), and other substances (two) were present in the volatile fractions of the samples. Moreover, three characteristic molecules (ethyl acetate, 1-octen-3-one, and caproic acid) increased their content as the fermentation time increased.

In contrast, Dan et al. [64] identified 53 and 43 volatile compounds from skimmed milk fermented by two LAB strains (*S. thermophilus* and *Lactobacillus delbrueckii* ssp. *bulgaricus*); these compounds were mainly 11 acids (e.g., C_2:0_, C_4:0_, C_6:0_–C_10:0_, benzoic acid, etc.), 10 aldehydes (e.g., nonanal, 2-methylundecanal, (*E*,*E*)-2,4-heptadienal, benzaldehyde, acetaldehyde, etc.), 13 ketones (e.g., 2-undecanone, 2-nonanone, 2-heptanone, acetoin, etc.), 13 alcohols (e.g., 1-nonanol, 2-nonanol, ethanol, etc.), seven esters (e.g., δ-nonalactone, ethyl caprate/caproate/butyrate, etc.), and 19 hydrocarbons (including terpenes, humulene, caryophyllene, C_11_–C_13_, xylene, toluene, etc.). The authors established that the volatile composition of the fermented milk product depended on the LAB strains (starter cultures) as well as whether they were used individually or in combination. Lastly, Shepard et al. [16] determined the volatile constituents (34) related to the aroma profiles of 32 commercial sour cream samples; as a result, 13 components (alkyl sulfides, ethyl(methyl)-alkenyl(alkyl)-ketones, aldehydes, SCFAs, etc.) were responsible for the flavor of 31–32 samples. Based on the concentrations of the 34 constituents, the increasing order of contribution to aroma was acetic acid > acetaldehyde > butyric acid > acetoin > octanal > 2-butanone > nonanal; furthermore, the authors stated that the profiled aroma compounds were derived (by biochemical reactions) from constituents inherent to the RM or its thermal treatment.

The comparison between the literature reports mentioned above and the results of this research showed relevant differences and some similarities; i.e., the volatile profiles of SC samples were mainly characterized by ethyl esters (as the most abundant constituents, e.g., ethyl acetate, ethyl caprate, etc.), followed by fatty (including short-chain (C_2:0_–C_5:0_ acids) and unsaturated) and aromatic acids, alcohols (ethanol, isoamyl alcohol, (2*E*)-tridecen-1-ol, etc.), sesquiterpenes, aliphatic aldehydes, and acetoin. Other contributing molecules were methyl alkyl ketones (2-pentadecanone, 2-tridecanone, 2-nonanone, etc.), lactones (δ-dodecalactone and δ-decalactone), and benzenethanol. This composition showed similarity with the reports by Dan et al. [64] and Zhao et al. [61] in specific constituents (C_10:0_, C_8:0_, benzoic acid, C_6:0_, C_4:0_, C_2:0_, nonanal, 2-methylundecanal, 2-undecanone, 2-nonanone, 2-heptanone, acetoin, 1-nonanol, 2-nonanol, ethanol, ethyl caprate, ethyl caproate, ethyl butyrate, humulene, caryophyllene, *n*C_11_–*n*C_13_ hydrocarbons, and toluene) but differences (in most chemical structures) with that reported by Wang et al. and Tan et al. [62,63]. Finally, the contrast between the composition determined by Shepard et al. [16] and this report only showed similarity in the content of acetoin and C_2:0_ and C_4:0_ acids.

All these compositional comparisons between SC and other similar dairy products based on milk fermentation (according to the available scientific literature) are necessary to support that the volatile chemical profile established for this artisanal product is original and unrepeatable, and to attribute to it one of the distinctive criteria that could give rise to its protected designation of origin.

On the other hand, it should be noted that the main metabolites identified in SC samples are probably the result of lipolysis and/or β-oxidation of fatty acids (free fatty acids, methyl alkyl ketones, alcohols, and lactones), as well as glycolysis [metabolism of lactose (ethanol and SCFAs) and citrate (ethyl acetate and acetoin)] carried out during milk fermentation by LAB [9,12,60,63]; thus, (i) the most representative constituents in the SC samples were ethyl esters (C_2_–C_12_, C_10:1_(Δ^9^)–C_18:1_(*cis*-Δ^9^), and C_13_–C_16_), which would contribute a floral and fruity note (or defect/off-flavor, depending on the concentration) to the SC [58,65], and (ii) according to the results on LAB isolation from the majority of SC samples (data not yet published by the coauthors, but used with permission), the predominant strain was *Limosilactobacillus fermentum* (along with *Lacticaseibacillus rhamnosus*), which could be responsible for the production of specific volatile compounds in SC; but also, as these strains were different from the LAB strains reported by the authors mentioned during the discussion, the SC volatiles could differ from the chemical profiles described by them in their fermented milk products.

Although the mechanism of ethyl ester biosynthesis by LAB in fermented milk has not been fully elucidated, it is hypothesized that the most likely route of biosynthesis could be via alcoholysis (by transferases) and/or hydrolysis (by esterases), depending on the strain involved in the fermentation, as well as the types of glycerides (acylglycerols) and alcohols [58,66,67]. In this manner, Liu et al. and Holland et al. [58,67] stated that a rate-limiting factor for ethyl ester formation in fermented products (e.g., cheeses) was the ethanol availability (>ethanol content > ethyl ester of fatty acid content), but also the type of glycerides (e.g., tri-, di-, and mono-) present in the milk, as well as the water content and activity. Thus, considering *L. fermentum* and *L. rhamnosus* (strains isolated in most of the SC samples), both strains prefer di- and mono-glycerides as substrates for alcoholysis, producing esters between C_6_–C_14_, mainly ethyl caprate (C_10:0_), ethyl caprylate (C_8:0_), and ethyl caproate (C_6:0_), as mentioned by Holland et al. and Liu et al.; hence, this could explain why ethyl esters (up to C_14:0_) were mostly identified in the SC samples.

Last but not least, *L. fermentum* could produce some chemical constituents in non-negligible amounts (~5%) that would confer a less-palatable perception to SC, e.g., acetic acid (vinegar-like taste) and acetoin (strong butter-like taste) [68]; the tastes [related to C_2:0_/acetoin, which were identified in the SC samples (1.0–3.9%/0.7–3.4%)] described above are characteristic notes of the SC product (acrid/sour and butter-like tastes, based on preliminary sensory testing—information not yet published, but used with permission).

As for the PCA and LDA applied to the data, they allowed finding and demonstrating close relationships between the volatile fractions of the SC samples as a function of the manufacturing process and locations, as well as determining those main structural groups responsible for the possible identity profile of the SC. The latter can be visualized in a general way with a box-plot representation (Figure 5), where through the comparison between matrices (RM and SC) based on volatile chemical components, for example, a decrease of FSFAs and an increase of ESTs (produced by the condensation/substitution reaction between an organic acid and an aliphatic alcohol) were observed. Nevertheless, it should be highlighted that locations 4 and 6 using process 4 (cooked whey residue from cheese + salting whey or commercial milk cream) along with location 5 using process 5 (rennet curdling) produced SC with the highest variability in the main volatile constituents identified for most SC samples, i.e., (i) the lowest and lower contents of FSFAs and esters for both L4 and L6, (ii) the highest and lowest contents of FSFAs (including SCFAs) and esters, respectively for L5, and (iii) the lowest (L4) and mid-range (L6) contents of SCFAs.

Of course, since the raw material for process 4 was whey (containing up to 14% protein and up to 4% fat [69]) from artisanal cheese making, its fat content must have been low and, consequently, lipolytic activity (or β-oxidation of fatty acids) by LAB during the fermentation process did not produce amounts of FSFAs and esters (derived from free fatty acids) comparable to the other SC samples, which would be in agreement with that reported by Asif et al. [70], who determined that during the buttermilk fermentation (by LAB) to produce cheddar cheese, the formation of free fatty acids by lipolysis depended on the fat content of the matrix, i.e., the higher the fat content, the higher the amount of free fatty acids. In addition, if there was less fat content (as well as low glyceride content), the esterification by LAB (alcoholysis) should also be low [58,67].

On the other hand, as it is well known, rennet is a complex mixture of protease-type enzymes (mainly chymosin and pectin) used industrially or domestically to curdle milk (casein coagulation) and produce fermented dairy foods (e.g., ripened and fresh cheeses), which might present desirable or undesirable sensory characteristics (taste and flavor) depending on storage time, as well as whether or not the rennet contains some lipases [71]. However, when Castillo et al. [72] studied the effect of the use of rennet (sanitized paste) on the volatile fraction of a hand-made cheese (from goat’s milk), they found the highest content of SCFAs (C_4:0_ >> C_6:0_), an increased content of mono-/di-glyceride, and a low content of ethyl esters (C_8:0_ and C_6:0_ esters being the majority) compared to other industrially processed cheeses; they also suggested that the high SCFA content was due to native lipases in raw milk. These findings showed similarities with the SC from L5: the highest content of SCFAs (differing in C_6:0_ >>> C_4:0_), an increase of monoglycerides (related to FSFA content), and a low content of ethyl esters (C_8:0_ > C_6:0_); consequently, the volatile chemical profile was different from that of the other SC samples.

Finally, Lytou et al. [73] described that (i) SPME and GC-MS are widely used techniques for food volatilome analysis, (ii) multivariate analysis (PCA and LDA) is an important tool for an accurate description of the obtained data, (iii) each food would have a particular volatile chemical fingerprint, and (iv) food volatilome profiles could be a useful and effective tool for the assessment of both quality and authenticity. According to these premises, the volatilome profile of SC could be established in this research and, therefore, this food would have its own volatile chemical fingerprint, which is described above.

## 4. Materials and Methods

### 4.1. Reagents, Standards and Materials

Dichloromethane (ACS grade, Alfa Aesar, Haverhill, MA, USA), BF_3_/methanol (14%, Ref. B1252, Sigma-Aldrich, Darmstadt, Germany), *n*-hexane (ACS grade, Merck, Billerica, MA, USA), α-pinene (98%, Ref. 147524, Sigma-Aldrich, Darmstadt, Germany), DL-limonene (for synthesis, Ref. 8.14546, Sigma-Aldrich, Darmstadt, Germany), camphor (>95% Merck), δ-decalactone (>98%, Ref. W236101, Sigma-Aldrich, Darmstadt, Germany), δ-dodecalactone (>97%, Ref. W240109, Sigma-Aldrich, Darmstadt, Germany), acetic acid glacial (>99%, Ref. 695092, Sigma-Aldrich, Darmstadt, Germany), butyric acid (>99%, Ref. B103500, Sigma-Aldrich, Darmstadt, Germany), valeric acid (>99%, Ref. 240370, Sigma-Aldrich), FAME Mix C_8_–C_24_ (Ref. 18918, Sigma Aldrich), C_10_–C_40_ alkane standard mixture (Ref. 68281, Supelco, Darmstadt, Germany), type I water (milli-Q^®^ Integral, Merck, Billerica, MA, USA), NaCl (≥99.5%, Merck, Billerica, MA, USA), SPME device (Supelco, Darmstadt, Germany), and coated fiber of divinylbenzene/carboxen/polydimethylsiloxane (DVB/CAR/PDMS, 50/30 μm) were used.

### 4.2. Collection of Raw Milk and Suero Costeño Samples

Eight representative samples of cow’s milk and suero costeño were collected from eight individual farms and four municipalities (mainly artisanal producers) in the department of Córdoba (northern region of Colombia—Table S20) based on convenience sampling, together with the stratified random sampling as described by Greenfield and Southgate (FAO) [74] for food composition data collection. In addition, farmers produced SC based primarily on certain factors: (i) from naturally fermented raw milk (37.5% collected samples), (ii) using boiled milk (25.0% collected samples), (iii) cooked residual whey from cheese preparation (25.0% collected samples), and (iv) rennet-fermented raw milk (12.5% collected samples). SC and RM samples were collected in the second half of 2022 (August to December) and, as current practice, the fermentation process was carried out for at least 24 h (with one exception, for 72 h). Then, 150 mL of each homogenized/representative sample was collected (in triplicate) in Falcon tubes and immediately taken to the chemical laboratory for processing and analysis. Some samples were temporarily stored at −20 °C until further processing and analysis; this storage condition was critical to decrease/prevent the biochemical activity of LAB or other microorganisms present in the SC and, therefore, did not alter their organoleptic (aroma and taste) and physicochemical properties [75,76,77].

### 4.3. Volatile Fractions

To isolate the volatile fractions from the samples, they were mixed with matrix modifiers, i.e., 10% NaCl (salting effect) for milk and water (1:2 H_2_O:sample) for SC [50,78]. Moreover, two sampling techniques were used: headspace solid-phase microextraction and simultaneous distillation-solvent extraction [79]. Each sample was extracted in triplicate by each technique.

For simultaneous distillation-solvent extraction, the volatile fractions of the dairy samples (10 g) were obtained by the Likens & Nickerson microscale apparatus, modified by Godefroot et al. [80], using CH_2_Cl_2_ (2 mL) as extraction solvent for 2 h. The extracts were dehydrated with anhydrous sodium sulfate and 1 μL of each of them (individually) was analyzed by GC-FID/MSD.

For headspace solid-phase microextraction, SC and RM vapor-phase constituents were trapped using a SPME device (Supelco) with a DVB/CAR/PDMS (50/30 μm) coated fiber, sampling the headspace. Food samples (10 g) were thermally pre-conditioned (45 °C) for 10 min and then the fiber was exposed to the headspace of each sample (separately) at 45 °C during 45 min (with magnetic stirring) as described by Gioacchini et al. [81], Zhang et al. [82], and Mounchili et al. [83]. After sampling was completed, the SPME fiber was desorbed (5 min, 250 °C) with the analytes inside the GC-FID/MS inlet port.

For GC-FID/MS analysis, each volatile fraction (SPME and SDE) was analyzed using a Trace 1310 gas chromatograph with a flame ionization detector (FID, 250 °C) and an ISQ Series mass spectrometer (Thermo Fisher Scientific), with split/splitless inlet (split ratio, 10:1—SDE; splitless—SPME), a liquid autosampler (AI/AS 1310 Series, Thermo Fisher Scientific), or manual injection (SPME). Separation of the mixtures was obtained (i) on a Rxi^®^-1MS column (crossbond PDMS, 30 m × 0.25 mm ID × 0.5 µm df, Restek, Bellefonte, PA, USA) and (ii) with linear temperature programming in the GC oven: 60 °C (5 min) @ 3 °C/min up to 250 °C (5 min). The separated components were reported as normalized area percentages (% relative amounts).

Mass spectra were obtained by electron ionization (EI, 70 eV) and a quadrupole mass analyzer with mass ranges *m*/*z* 40–350, in full scan mode; helium (99.999%) was used as the carrier gas (1.0 mL/min) and the temperatures of the transfer line and ion source were set to 250 °C and 230 °C, respectively. Chromatographic and spectroscopic data were processed using Thermo Xcalibur^TM^ (Version 2.2 SP1.48, Thermo Fisher Scientific) and Automated Mass Spectral Deconvolution and Identification System (AMDIS, Build 130.53, Version 2.70) software.

### 4.4. Isolation of Fats and Their Fatty Acids

Crude fat was determined (for RM and SC samples) using the modified Rose-Gottlieb method—AOAC official method 932.02-1945 [84] and ISO 23318 IDF 249 [85].

Fatty acid profiles were determined based on the AOAC official method 996.06 [fat (total, saturated, and unsaturated) in foods (hydrolytic extraction and GC-MS analysis)] [86], AOAC official method 969.33 (fatty acids in oils and fats preparation of methyl esters) [87], and Ackman [88]. The free fatty acids resulting from fat hydrolysis were derivatized to fatty acid methyl esters (FAMEs) using BF_3_/CH_3_OH and extracted with hexane. The FAMEs of the samples were confirmed by comparison with retention times and indices, and the mass spectra of FAME Mix C_8_–C_24_ (Table S24).

For GC-FID/MS analysis, each FAME extract was also analyzed by the same separation and detection systems (GC-FID/MS) described above, with a 1:10 split ratio. Separation of the mixtures was obtained with a GC oven temperature program that started at 100 °C (2 min) @ 2 °C/min up to 150 °C (5 min), @ 3 °C/min up to 240 °C (2 min), and @ 6 °C/min up to 280 °C (2 min). Additionally, mass spectra were obtained on a quadrupole analyzer with mass ranges of *m*/*z* 45–450. The other operating conditions of the mass detector as well as data processing were the same as described above. The separated FAMEs were reported as normalized area percentages (% relative amounts).

### 4.5. Identity of the Chemical Constituents Analyzed by GC-MSD

The components were identified by comparing mass spectra and calculated retention indices (R_I_) with available libraries (NIST11, NIST Retention Index, and Wiley9) and existing scientific literature [32,89,90,91,92,93,94]. As a complement, certified standards (lactones, terpenes, FAMEs, and linear hydrocarbons) were used to confirm some constituents. Linear retention indices were previously calculated in the usual way (C_10_–C_40_ aliphatic hydrocarbon series) for both GC-FID and GC-MSD analysis.

### 4.6. Design of Experiment

The selection of the SC samples (eight) was performed in a stratified random sampling (SRS), considering the most important (or common) manufacturing methods (Table S20), as well as the different localities where SC was prepared in an artisanal (non-industrial) way by small- and medium-sized farmer associations in the region. The number of samples was obtained by calculations from the Australian Bureau of Statistics website [95] considering a total of 11 samples, a 95% confidence level and a confidence intervale of 0.1; the standard and relative standard errors were, respectively, 0.051 and 5.67 (Figure S5). Subsequently, samples were also selected based on the manufacturing process using both SRS and convenience sampling (Table S25), as suggested by FAO [74]. Lastly, milk samples were selected by simple random sampling, taking four samples from the eight locations where the farms of the small- to medium-sized peasant associations were located.

### 4.7. Statistical Analysis

The raw data of the results were treated by non-parametric ANOVA (Kruskal–Wallis and Friedman tests), together with the Shapiro–Wilks test (normality of data (*p* <0.05)) and inference based on two samples (F test for equal variances). All data acquired from the samples were statistically treated and subjected to principal component analysis (PCA) and linear discriminant analysis (LDA) as tools of multivariate statistical analysis using the InfoStat software (free version 2020).

## 5. Conclusions

From this research, it was possible to conclude that (i) the chemical nature related to fatty acids (composition and relative amount of FAs) of RM was preserved in all SC samples analyzed; moreover, this food was a source of MUFAs, n-3, and n-6 and had acceptable values of AI and TI; (ii) the manufacturing process of SC influenced the composition of the volatile fractions as demonstrated by the variability of some of its constituents; however, the chemical volatile profiles with negligible variabilities were found in the SC prepared using raw milk and spontaneous fermentation and, in consequence, it is advisable to standardize the process (without affecting the characteristic of the artisanal product), as well as to implement good manufacturing practices; and last of all, (iii) the principal types of molecules (differentiators) that would support the possible establishment of chemical identity for this food product (for 75–88% of the SC samples) were ethyl esters [C_2_–C_12_ and C_10:1_(Δ^9^)–C_18:1_(*cis*-Δ^9^)], aliphatic alcohols [(2*E*)-tridecen-1-ol, ethanol, and isoamyl alcohol], aldehydes (palmitaldehyde and myristylaldehyde), methyl alkyl ketones (2-pentadecanone, 2-tridecanone, 2-undecanone, and 2-nonanone), sesquiterpenes (β-caryophyllene, α-humulene, and selinane), monoterpenes (limonene), benzoic acid, and benzenethanol, along with SCFAs and acetoin (resulting from the spontaneous fermentative process).

## Data Availability

The most important data are included in the Supplementary Materials, as well as Appendix A.

## Supplementary Materials

The following supporting information can be downloaded at: https://www.mdpi.com/article/10.3390/molecules30122524/s1, Table S1: Result of Shapiro–Wilks test (modified) for fat content yields of RM and SC samples; Table S2: Result of non-parametric ANOVA using Friedman’s test for fat content yields of RM and SC samples; Table S3: Result of non-parametric ANOVA using the Kruskal–Wallis test for fat content yields of RM and SC samples; Figure S1: FAME profiles by GC-MS of a suero costeño sample (location 1); Table S4: Identification and content (>0.4%) of fatty acids in samples of raw milk and suero costeño from eight locations of Córdoba (Colombia); Figure S2: Distribution of the four main FAs that constituted the RM and SC samples from the eight locations studied; Table S5: Result of Shapiro–Wilks test (modified) for major fatty acids of RM and SC samples; Table S6: Result of non-parametric ANOVA using Friedman’s test for major fatty acids of RM and SC samples; Table S7: Result of non-parametric ANOVA using the Kruskal–Wallis test for major fatty acids of RM and SC samples; Table S8: Result of Shapiro–Wilks test (modified) for the content of fatty acids by compound families of RM and SC samples; Table S9: Result of non-parametric ANOVA using Friedman’s test for the content of fatty acids by compound families of RM and SC samples; Table S10: Result of non-parametric ANOVA using the Kruskal–Wallis test for the content of fatty acids by compound families of RM and SC samples; Table S11: Result of Shapiro–Wilks test (modified) for AI and TI values of RM and SC samples; Table S12: Result of non-parametric ANOVA using Friedman’s test for AI and TI values of RM and SC samples; Table S13: Result of non-parametric ANOVA using the Kruskal–Wallis test for AI and TI values of RM and SC samples; Figure S3: Volatile profiles by GC-MS of the raw milk (location 1) by (A) SDE and (B) HS-SPME; Figure S4: Volatile profiles by GC-MS of the suero costeño (location 1) by SDE (A) and HS-SPME (B); Table S14: Result of non-parametric ANOVA using the Kruskal–Wallis test for the volatile fractions by SDE and HS-SPME of raw milk samples; Table S15: Result of non-parametric ANOVA using Friedman’s test for the volatile fractions by SDE and HS-SPME of raw milk samples; Table S16: Result of non-parametric ANOVA using Friedman’s test comparing the chemical composition (average relative amount) of volatile fractions by SDE and HS-SPME of raw milk samples; Table S17: Result of non-parametric ANOVA using the Kruskal–Wallis test comparing the chemical composition (average relative amount) of volatile fractions by SDE and HS-SPME of raw milk samples; Table S18: Result of non-parametric ANOVA using Friedman’s test for the volatile fractions by SDE and HS-SPME of the suero costeño samples; Table S19: Result of non-parametric ANOVA using the Kruskal–Wallis test for the volatile fractions by SDE and HS-SPME of suero costeño samples; Table S20: Sampling places (eight) and manufacturing process of SC in four municipalities of Córdoba (Colombia); Table S21: F Test for equality of variances comparing volatiles between raw milk and suero costeño; Table S22: Chemical composition of volatile fractions by SDE/GC-FID/MSD of raw milk and suero costeño samples classified according to the type of compound identified; Table S23: Chemical composition of volatile fractions by HS-SPME/GC-FID/MSD of raw milk and suero costeño samples classified according to the type of compound identified; Table S24: Composition of FAME Mix C_8_–C_24_ (Ref. 18918. Sigma Aldrich) determined by GC-FID/MSD; Figure S5: Determination of sample size for SC using sample size calculator (Australian Bureau of Statistics); Table S25: Selection of samples for chemical analysis based on stratified random sampling.

## Appendix A

Table A1. Identification of constituents (>0.1%) present in the volatile fractions by SDE of the raw milk and suero costeño samples from eight locations of Córdoba (Colombia). Table A2. Identification of constituents (>0.1%) present in the volatile fractions by HS-SPME of the raw milk and suero costeño samples from eight locations of Córdoba (Colombia).

**Table A1:** Identification of constituents (>0.1%) present in the volatile fractions by SDE of the raw milk and suero costeño samples from eight locations of Córdoba (Colombia).

Peak	Constituents	R_Ical_	R_Ilit_	Raw Milk ^‡^				Suero Costeño ^‡^							
				L1	L3	L4	L7	^a^ L1	^a^ L2	^a^ L3	^b^ L4	^c^ L5	^b^ L6	^a^ L7	^a^ L8
1	3-Furanmethanol	843	835	----	----	----	----	----	----	----	0.1	----	----	----	----
2	Isovaleric acid—C_5:0_	849	851	----	0.9	----	----	0.4	0.2	0.09	0.1	0.2	4.2	0.08	0.3
3	2-Furanmethanol	852	849	----	----	----	----	----	----	----	0.1	----	----	----	0.04
4	2-Methylbutanoic acid—C_5:0_	860	861	----	0.6	----	----		0.5	0.4	----	0.4	0.6	0.4	0.4
5	Xilene ^†^	869	865	----	----	----	----	----	----	----	0.1	----	----	----	----
6	5-Methyl-2-hexanone	870	865	0.4	0.6	----	0.2	0.2	0.2	0.1	0.5	0.2	1.5	0.2	0.6
7	Valeric acid ^†^—C_5:0_	870	880	----	0.5	----	----	0.3	0.3	0.2	----	0.5	0.8	0.3	0.6
8	2-Heptanone	877	880	0.4	0.6	----	0.2	0.6	0.1	0.1	1.1	0.2	1.8	0.3	1.2
9	Styrene	882	887	----	----	----	----	----	0.06	0.2	----	0.1	0.6	----	0.1
10	Furfuryl ethyl ether derivative *	893	----	----	----	----	----	----	0.2	0.1	----	----	----	----	----
11	Tricyclene	919	920	----	----	----	----	----	----	----	----	0.2	----	0.08	0.2
12	α-Pinene ^†^	931	930	----	----	----	----	----	0.03	0.1	02	0.06	----	0.2	0.3
13	Fenchene	940	942	----	----	----	0.07	trace	0.04	0.1	0.2	0.1	----	0.2	0.3
14	Camphene	945	947	----	----	----	0.2	trace	0.05	0.2	0.2	0.3	----	0.2	0.6
15	Caproic acid—C_6:0_	981	983	9.0	13.3	5.9	0.8	8.8	6.2	4.2	1.4	27.3	2.0	5.6	10.9
16	Ethyl caproate	987	985	----	----	----	----	0.7	0.4	1.2	----	----	----	1.2	0.7
17	Decane ^†^	1000	1000	----	----	0.1	0.07	----	----	----	0.09	0.02	----	----	----
18	*p*-Cymene	1016	1015	----	----	----	----	----	----	0.1	----	----	----	----	0.3
19	Limonene ^†^	1020	1022	----	1.5	0.3	0.1	----	0.1	0.1	----	----	0.3	----	0.4
20	2,6-Dimethylnonane	1024	1025	0.3	1.0	0.4	0.2	----	0.04	0.1	0.3	----	----	0.1	----
21	*m*-Tolualdehyde	1053	1056	0.6	----	----	----	0.05	0.1	0.07	----	----	0.4	0.1	0.1
22	2-Methyl-decane	1064	1065	1.4	1.6	1.2	0.6	----	0.1	0.2	0.6	0.09	----	0.4	0.08
23	4-Methylhexanoic acid	1065	----	----	----	----	----	----	----	----	----	0.1	----	----	----
24	2,5-Dimethylethylbenzene	1066	1067	----	----	----	----	----	----	----	----	----	----	----	0.1
25	*p*-Cresol	1068	1066	----	----	0.3	----	----	----	----	----	----	----	----	----
26	2-Nonanone	1072	1070	0.4	0.5	----	0.4	0.4	0.2	0.2	1.0	0.2	1.9	0.4	1.0
27	Enanthylic acid—C_7:0_	1074	1073	----	----	0.2	----	----	trace	----	----	0.06	----	----	----
28	Pelargonaldehyde	1085	1084	----	----	----	----	0.1	----	----	0.1	0.02	----	----	----
29	Benzenethanol	1093	1091	0.3	0.4	0.4	----	1	1.8	0.5	0.2	0.8	0.5	1.8	0.4
30	Undecane ^†^	1100	1100	0.9	0.2	0.2	0.2	----	0.07	----	0.2	----	----	0.2	----
31	Camphor ^†^	1121	1125	----	----	----	0.04	----	trace	----	----	----	----	----	0.06
32	Benzoic acid ^†^	1178	1174	1.5	1.4	2.4	0.8	2.2	1.4	0.8	1.7	2.3	0.8	2.4	1.9
33	Ethyl caprylate	1184	1180	----	----	----	----	1.7	2.4	3.3	0.2	----	----	3.3	3.7
34	Caprylic acid—C_8:0_	1187	1180	13.6	15.0	15.7	12.2	8.9	10.5	8.3	2.3	11.8	2.5	9.6	12.7
35	1-Dodecene	1189	1187	----	0.3	----	----	----	----	----	----	----	----	----	----
36	(3*Z*)-Dodecene	1194	1195	0.2	----	----	0.2	----	----	----	----	----	----	----	----
37	Dodecane ^†^	1200	1200	----	0.4	0.1	0.09	----	0.08	----	----	----	----	0.08	----
38	4,8-Dimethylundecane	1211	1212	0.3	0.3	0.1	0.09	----	----	----	----	----	----	----	----
39	2,6-Dimethylundecane	1218	1216	----	----	----	----	----	----	----	0.1	----	----	0.09	----
40	2,8-Dimethylundecane	1225	1221	----	----	----	----	----	----	----	0.1	----	----	0.07	----
41	β-Phenethyl acetate	1235	1233	----	----	----	----	0.03	0.02	----	0.02	----	----	0.03	0.07
42	Isoamyl caproate	1239	1234	----	----	----	----	----	----	----	----	0.06	----	----	----
43	δ-Octalactone	1244	1243	0.04	----	----	0.03	0.04	0.04	----	0.09	0.04	0.08	0.04	0.05
44	^d^ 1,3-Di-tert-butylbenzene	1245	1245	0.9	1.2	0.8	0.4	0.9	----	----	0.5	0.1	3.5	0.3	0.04
45	(2*Z*)-Decenal	1246	1249	----	----	----	----	0.02	0.02	----	----	0.01	----	----	----
46	Pelargonic acid—C_9:0_	1273	1272	0.2	1.0	0.3	0.2	0.2	0.2	0.3	----	0.2	0.4	0.2	0.2
47	2-Undecanone	1281	1279	0.6	0.4	trace	0.2	0.5	0.3	0.2	1.6	0.3	2.8	0.3	1.2
48	Ethyl pelargonate	1285	1282	----	----	0.1	----	----	0.02	0.04	----	----	----	0.02	0.04
49	2,6,11-Trimethyldodecane	1286	----	1.2	1.4	0.8	0.5	----	0.05	0.2	0.7	0.09	----	0.4	0.06
50	Tridecane ^†^	1300	1300	0.2	0.3	0.2	0.1	----	----	----	0.1	----	----	0.05	----
51	4,6-Dimethyldodecane	1338	----	0.7	0.9	0.5	0.3	----	0.04	0.05	0.4	0.06	1.9	0.2	----
52	Isobutyl caprylate	1338	1334	----	----	----	----	----	0.01	0.03	----	----	----	----	----
53	(7*Z*)-Tetradecene	1362	1365	----	----	----	----	----	----	----	0.3	0.4	0.3	----	0.6
54	Caproleic acid—C_10:1_	1376	1362	2.6	3.5	1.6	2.1	0.2	0.09	0.8	0.8	1.6	----	0.1	2.0
55	Ethyl 9-decenoate	1377	1370		----	----	----	0.9	1.6	1.6	2.2	----	0.2	1.5	2.8
56	Ethyl caprate	1385	1381	0.2	----	----	----	4.8	6.3	6.8	0.3	----	0.2	7.4	5.7
57	Capric acid—C_10:0_	1393	1388	25.4	17.1	32.3	45.7	16.2	20.8	17.6	6.6	20.0	5.0	16.7	13.7
58	Dodecanal	1393	1390	0.3	0.5	0.2	----	0.1	----	----	----	----	0.9	----	----
59	Tetradecane ^†^	1400	1400	0.2	0.4	0.07	0.06	0.1	0.1	----	0.6	0.2	0.5	0.1	0.5
60	Cyperene	1402	1404	----	----	----	----	0.06	----	----	----	0.1	0.1	----	----
61	β-Caryophyllene ^†^	1422	1425	----	0.2	0.1	0.4	0.2	0.4	0.3	3.4	0.3	0.7	0.7	2.3
62	2-Methylbutyl octanoate	1442	1444	----	----	----	----	----	0.07	0.03	----	----	----	----	----
63	α-Humulene	1455	1452	----	0.2	0.09	0.2	0.2	0.3	0.2	2.6	0.4	0.5	0.4	1.6
64	δ-Decalactone ^†^	1464	1463	0.7	0.3	0.1	0.08	0.4	0.3	0.20	0.9	0.02	0.9	0.3	0.3
65	2,6,10-Trimethyltridecane	1470	1467	0.5	0.4	0.2	0.1	----	0.04	0.1	0.3	----	1.0	0.1	0.1
66	Undecanoic acid—C_11:0_	1470	1469	----	----	0.1	0.2	0.08	----	0.6	----	0.06	----	0.08	0.1
67	(2*E*)-Dodecenal	1472	1472	----	----	----	----	----	0.1	----	----	----	----	----	----
68	Propyl decanoate	1483	1481	----	----	----	----	----	----	----	----	----	----	----	0.08
69	2-Tridecanone	1485	1482	0.8	0.5	0.04	0.2	0.7	0.5	0.3	3.3	0.4	4.0	0.5	1.2
70	Ethyl undecanoate	1487	1483	----	----	----	----	----	----	0.1	----	----	----	----	----
71	Selinane *	1489	----	----	----	----	0.09	----	0.08	0.1	1.4	0.2	----	0.1	0.6
72	Pentadecane ^†^	1500	1500	0.9	1.0	0.5	0.4	0.08	0.1	0.09	0.9	0.1	2.7	0.3	0.2
73	^d^ 2,4-Di-tert-butylphenol	1509	1512	0.6	0.9	0.3	0.2	0.2	0.05	0.09	0.6	0.07	1.6	0.3	0.06
74	γ-Cadinene	1513	1512	----	----	----	----	----	0.03	0.08	0.3	0.02	----	0.1	0.07
75	δ-Cadinene	1521	1522	----	----	----	----	----	0.05	trace	0.5	0.03	----	0.1	0.2
76	Isobutyl caprate	1538	1533	----	----	----	----	----	0.03	----	----	0.01	----	0.04	----
77	2,6,10-Trimethyltetradecane	1554	1557	0.8	0.8	0.4	0.2	0.04	0.05	0.2	0.5	0.08	1.8	0.2	0.07
78	(2*E*)-Tridecen-1-ol	1565	1564	----	----	----	----	1.4	0.7	1.1	3.1	1.2	5.5	0.8	1.9
79	Lauric acid—C_12:0_	1573	1570	10.0	7.1	10.7	15.2	9.6	11.9	9.5	5.9	10.5	4.1	8.0	5.4
80	(5*Z*)-Dodecenoic acid—C_12:1_	1580	1578	----	----	----	----	----	0.3	----	----	----	----	0.2	0.3
81	Ethyl laurate	1585	1583	----	----	0.1	----	2.4	4.5	6.7	0.5	0.2	0.3	3.5	2.7
82	Tridecan-1-ol	1593	1586	0.2	0.1	0.1	0.04	----	0.02	----	0.1	----	0.3	0.09	----
83	Hexadecane ^†^	1600	1600	0.2	0.2	----	----	----	----	----	0.2	0.05	0.4	0.09	0.09
84	Myristylaldehyde	1603	1601	0.1	0.2	----	0.04	0.3	0.2	0.09	1.7	0.2	1.3	0.2	0.3
85	Phenylethyl caproate	1620	1619	----	----	----	----	----	----	----	----	0.04	----	----	----
86	Isoamyl n-decanoate	1637	1633	----	----	----	----	----	0.1	0.1	----	----	----	----	----
87	Myristic alcohol	1641	1647	----	----	----	----	----	----	----	0.9	----	0.1	0.08	0.1
88	2-Methylbutyl decanoate	1643	1647	----	----	----	----	----	0.04	0.1	----	----	----	0.04	----
89	γ-Dodecalactone	1646	1645	0.06	0.06	----	0.02	----	trace	trace	trace	0.04	0.4	tr	0.06
90	Tridecanoic acid—C_13:0_	1660	1659	----	----	----	----	0.1	0.1	0.1	0.2	0.1	----	0.1	0.1
91	δ-Dodecalactone ^†^	1674	1675	1.1	0.6	0.1	0.2	1.1	0.6	0.4	1.6	0.06	1.0	0.5	0.3
92	13-Methyltetradecanal	1677	1680	----	----	----	----	----	----	----	2.4	0.2	2.0	0.2	0.3
93	Ethyl tridecanoate	1688	1689	----	----	0.05	----	----	0.08	0.3	----	----	----	0.2	0.05
94	2-Pentadecanone	1693	1698	0.6	0.3	0.1	0.1	0.7	0.7	0.4	3.8	0.4	3.7	0.5	0.6
95	Heptadecane ^†^	1700	1700	0.4	0.5	0.2	0.1	0.1	0.08	0.2	0.6	0.08	1.2	0.2	0.1
96	Pentadecanal	1703	1701	0.1	----	----	----	0.2	0.05	----	1.1	0.09	0.7	0.08	0.1
97	Myristoleic acid—C_14:1_ *	1752	----	0.9	0.3	1.3	1.0	0.4	0.4	0.3	0.3	1.1	0.4	0.3	0.6
98	Myristic acid—C_14:0_	1766	1763	4.7	1.9	8.5	6.0	11.7	7.6	4.6	4.7	7.8	5.6	5.1	4.2
99	Ethyl (9*Z*)-tetradecenoate	1772	1779	----	----	----	----	----	1.0	3.4	1.1	----	0.4	0.9	1.0
100	(9*Z*)-Hexadecenal	1780	1780	----	----	----	----	0.4	----	----	0.2	----	----	0.08	----
101	Ethyl myristate	1784	1780	----	----	----	----	3.6	4.8	8.6	0.6	0.3	0.3	4.1	2.1
102	Octadecene	1796	1797	0.5	0.7	0.2	0.2	0.1	0.2	0.2	1.4	0.2	1.9	0.4	0.2
103	Octadecane ^†^	1800	1800	----	----	----	----	----	0.09	----	0.4	----	----	----	----
104	Palmitaldehyde	1803	1807	0.3	0.3	0.05	0.03	1.3	0.2	0.3	4.3	0.3	2.1	0.4	0.3
105	Phytane	1819	1816	----	----	----	----	----	0.1	----	0.3	----	----	----	----
106	Isopentyl laurate	1834	1829	----	----	----	----	----	----	----	----	0.07	----	----	----
107	Neophytadiene	1841	1842	----	0.1	----	0.05	0.06	0.2	----	0.6	0.07	0.2	0.2	0.09
108	3,7,11,15-Tetramethylhexadec-2-ene	1851	----	0.3	0.6	----	0.1	0.2	0.3	0.1	1.7	0.2	2.0	0.4	0.4
109	Pentadecanoic acid—C_15:0_	1861	1860	0.2	----	0.2	0.2	0.6	----	0.1	----	0.3	----	0.08	0.07
110	Hexadecanol	1873	1875	----	----	----	0.2	0.06	0.05	----	0.3	0.03	0.6	0.3	----
111	(8*Z*)-14-Methyl-hexadecen-1-ol *	1881	----	----	----	0.1	----	0.1	0.02	----	0.3	0.02	0.3	0.07	0.02
112	Ethyl pentadecanoate	1888	1890	----	----	----	0.02	0.3	0.3	0.5	----	0.02	0.1	0.3	0.2
113	δ-Tetradecalactone *	1887	----	0.4	0.2	0.2	0.07	0.3	0.07	0.1	0.7	0.04	0.5	0.08	0.05
114	Nonadecane ^†^	1900	1900	----	----	0.1	----	----	----	----	----	----	----	0.1	----
115	Heptadecanal	1903	1898	----	0.2	----	----	0.04	----	----	0.3	----	----	----	----
116	Palmitoleic acid—C_16:1_	1932	1933	----	----	0.2	0.2	0.2	0.2	0.1	----	0.1	0.3	----	----
118	Methyl 2-methylpalmitate	1946	1944	----	----	----	----	0.1	----	0.03	----	----		0.06	----
119	(11*Z*)-Hexadecenoic acid—C_16:1_	1952	1953	----	----	----	----	----	----	----	----	0.05	2.1	----	----
120	Palmitic acid—C_16:0_	1961	1963	1.6	0.4	1.8	1.0	4.8	1.5	0.9	1.6	1.6	----	1.2	0.6
121	Ethyl (9*Z*)-hexadecenoate	1958	1955	----	----	----	----	----	0.4	1.8	----	----	----	0.5	0.2
122	(9*Z*)-Octadecenal *	1977	----	----	----	----	----	0.2	0.03	0.09	0.2	0.06	----	----	0.03
123	Ethyl palmitate	1982	1980	----	----	----	----	2.8	2.2	2.8	0.1	0.1	0.2	2.2	0.6
124	Octadecanal	2004	2002	0.2	0.2	0.1	0.06	0.3	0.05	trace	0.7	0.08	0.5	0.1	0.05
125	Isopropyl palmitate	2015	2017	----	----	----	----	----	----	----	----	0.02	----	----	----
126	Ethyl (9*Z*)-heptadecenoate *	2058	----	----	----	----	----	0.07	0.04	0.1	----	----	----	0.07	0.03
127	Octadecanol	2073	2074	0.2	----	----	0.2	----	0.05	----	0.6	0.03	0.5	0.3	----
128	δ-Hexadecalactone *	2100	----	0.09	----	----	----	0.07	0.02	trace	----	----	----	----	----
129	Heneicosane	2100	2100	----	----	----	----	0.03	----	0.1	----	----	----	0.1	----
130	(9*Z*)-Oleic acid—C_18:1_	2123	2116	0.2	----	0.07	0.1	0.2	0.1	0.1	----	0.09	----	----	----
131	Ethyl linoleate	2142	2140	----	----	----	----	0.1	0.08	0.2	----	----	----	0.1	0.03
132	Ethyl α-linolenate	2146	2145	----	----	----	----	0.1	0.06	0.2	----	----	----	0.1	----
133	Ethyl (9*Z*)-oleate	2150	2149	----	----	----	----	1.2	0.7	1.4	----	0.07	----	1.1	0.4
134	Ethyl elaidate	2165	----	----	----	----	----	0.2	0.09	0.3	----	----	----	0.2	0.03
135	Ethyl stearate	2180	2181	----	----	----	----	0.3	0.1	0.2	----	----	----	0.2	0.03
136	Ethyl (9*Z*,11*E*)-octadecadienoate *	2187	----	----	----	----	----	0.08	0.04	0.1	----	----	----	0.1	0.02
137	1-Docosene	2196	2195	0.3	0.3	0.2	0.08	----	0.02	0.09	0.3	0.04	1.0	0.2	0.03
Parcial sum				89.6	85.3	91.0	93.5	97.2	97.2	96.8	83.0	96.3	88.5	92.6	94.7
Unidentified constituents															
Branched hydrocarbons C_10_–C_26_				9.2	13.6	6.9	4.6	0.6	0.4	0.9	5.8	0.6	6.8	2.1	0.1
Branched aliphatic alcohols C_11_				----	----	----	----	0.2	----	----	----	0.1	----	----	----
Sesquiterpene hydrocarbons (C_15_H_24_)				----	----	----	----	----	----	----	----	----	----	----	0.2
Sesquiterpene hydrocarbons (C_15_H_26_)				0.0	0.3	0.3	0.5	0.4	0.6	0.1	6.5	1.4	2.0	0.8	3.7
Branched saturated fatty acids C_12_–C_15_				0.6	0.3	0.5	0.8	1.0	0.6	0.5	0.8	1.3	0.2	0.4	0.5
Branched unsaturated fatty acids C_12_–C_19_				0.1	0.1	1.3	0.1	----	----	----	0.2	0.2	0.3	0.08	----
Ethyl ester of fatty acid C_12_–C_16_				0.2	----	----	----	0.6	0.8	1.5	0.1	----	0.4	0.8	0.6
Branched aliphatic aldehydes C_15_				0.1	0.3	----	----	0.4	0.2	0.2	2.2	0.2	1.8	0.2	0.2
Branched hydrocarbons C_10_–C_26_				9.2	13.6	6.9	4.6	0.6	0.4	0.9	5.8	0.7	6.8	2.1	0.1
Parcial sum				10.3	14.5	9.0	6.0	3.1	2.5	3.2	15.6	4.0	11.5	4.3	5.3
Total (%)				99.9	99.8	100.0	99.5	100.0	99.7	100.0	98.7	100.0	100.0	96.9	100.0

^‡^ Area normalization (%) based on GC-FID analysis and calculated from the average value (mean ± sd) of two measurements. The standard deviation of each identified component ranges between 0.01 (mean values ≤ 1%)–0.8 (mean values ≤ 45.7%); different superscript letters (a–c) show significant differences in chemical compositions (*p* <0.05). R_I-Cal_: retention index calculated; R_I-it_: retention index of literature; ^†^ compared to the respective certified reference standard (R_t_, R_I_, and mass spectrum); ^d^ contaminant; * tentatively identified. L1: location 1—Cereté 5; L2: location 2—Chinú 2; L3: location 3—Chinú 3; L4: location 4—Chinú 4; L5: location 5—Ciénaga de Oro 4; L6: location 6—Ciénaga de Oro 8; L7: location 7—Sahagún 1; and L8: location 8—Sahagún 3.

**Table A2:** Identification of constituents (>0.1%) present in the volatile fractions by HS-SPME of the raw milk and suero costeño samples from eight locations of Córdoba (Colombia).

Peak	Constituents	R_Ical_	R_Ilit_	Raw Milk ^‡^				Suero Costeño ^‡^							
				L1	L3	L4	L7	^a^ L1	^a^ L2	^a^ L3	^b^ L4	^c^ L5	^b^ L6	^a^ L7	^a^ L8
1	Acetone ^†^	----	471	2.5	0.7	0.3	2.3	----	----	----	----	0.07	----	----	----
2	Ethanol ^†^	----	482	----	----	----	----	0.9	3.3	2.2	0.6	0.2	1.1	2.4	2.5
3	Ethyl acetate ^†^	----	615	----	----	----	----	0.8	3.8	7.2		0.6	3.0	1.8	3.5
4	Diacetyl	----	616	----	----	----	----	----	----	----	----	----	2.0	----	----
5	Acetic acid ^†^—C2:0	----	646	8.2	0.7	0.6	----	2.2	3.9	1	1.1	----	1.0	2.4	2.4
6	Acetoin	727	717	----	0.1	0.2	----	1.4	0.7	1.2	3.4	0.8	2.8	1.3	1.4
7	Isoamyl alcohol	749	741	1.3	5.0	0.5	----	2.5	2.9	2.7	1.9	1.5	2.8	1.4	1.4
8	Isobutyric acid—C_4:0_	754	752	----	----	----	----	----	----	0.2	----	----	1.5	----	----
9	Pentanol	757	752	1.3	0.7	0.7	----	----	1.0	----	2.7	----	2.7	----	2.1
10	Butyric acid—C_4:0_	776	775	1.4	0.8	0.3	0.6	0.8	0.4	0.2	----	1.6	1.3	0.5	0.6
11	Toluene	778	771	1.3	0.04	0.3	0.3	----	----	----	----	----	----	0.4	----
12	Ethyl butyrate	784	785	----	----	----	----	----	1.1	0.7	----	----	0.7	----	0.7
13	Isovaleric acid—C_5:0_	849	851	----	0.3	----	----	----	0.2	trace	----	----	0.5	----	0.3
14	2-Furanmethanol	852	849	----	----	----	----	----	----	----	----	----	0.4	----	----
15	2-Methylbutanoic acid—C_5:0_	860	861	----	0.2	----	----	----	----	----	----	----	0.4	----	0.2
16	5-Methyl-2-hexanone	870	865	1.4	0.3	0.07	0.4	0.2	----	0.2	0.8	0.1	1.3	----	0.04
17	Valeric acid ^†^—C_5:0_	870	880	----	0.3	----	----	----	0.07	----	----	----	0.4	----	----
18	2-Heptanol	873	877	----	----	----	----	----	----	0.4	----	----	1.6	----	----
19	2-Heptanone	877	880	2.0	0.3	0.08	0.8	0.2	----	----	1.3	0.09	0.4	----	----
20	Styrene	882	887	3.1	0.3	0.07	0.7	0.2	0.4	0.4	0.4	----	----	----	0.05
21	2,6-Dimethyl-4-heptanol	913	----	----	1.8	----	----	----	----	----	----	----	----	----	----
22	Tricyclene	919	920	----	----	----	0.1	0.02	----	----	0.4	0.05	----	----	0.05
23	Benzaldehyde	920	923	----	----	----	----	0.03	----	----	----	----	----	----	----
24	α-Pinene ^†^	931	930	----	----	----	0.2	----	----	0.04	1.2	0.2	0.3	0.1	0.2
25	Fenchene	940	942	----	----	----	0.4	----	----	----	----	----	----	----	----
26	Camphene	945	947	----	----	----	0.4	0.06	0.07	0.1	0.1	----	----	----	----
27	6-Methyl-2-heptanone	961	957	----	----	----	0.07	----	----	----	----	----	----	----	----
28	Caproic acid—C_6:0_	981	983	5.8	8.6	3.7	4.2	6.6	4.6	1.8	2.9	16.5	14.1	4.2	5.9
29	Ethyl caproate	987	985	----	----	0.7	----	0.7	0.7	0.9	----	1.0	0.8	1.6	2.4
30	Decane ^†^	1000	1000	----	----	0.1	0.07	----	----	----	0.09	0.02	----	----	----
31	α-Tolualdehyde	1014	1012	----	1.2	----	----	----	0.2	----	----	----	----	----	----
32	*p*-Cymene	1016	1015	----	----	----	----	----	----	0.1	----	----	----	----	0.3
33	Limonene ^†^	1020	1022	----	1.5	0.3	0.1	----	0.1	0.1	----	----	0.3	----	0.4
34	Isoamyl butyrate	1041	1040	0.3	1.0	0.4	0.2	----	0.04	0.1	0.3	----	----	0.1	----
35	*m*-Tolualdehyde	1053	1056	0.6	----	----	----	0.05	0.1	0.07	----	----	0.4	0.1	0.1
36	8-Nonen-2-one	1057	1055	----	----	----	1.3	----	----	----	----	----	----	----	----
37	Octan-1-ol	1058	1060	0.2	----	----	0.1	0.09	----	----	----	----	0.3	----	----
38	2-Methyl-decane	1064	1065	0.4	----	----	----	----	----	----	----	0.03	----	----	----
39	*p*-Cresol	1068	1066	----	0.09	0.02	----	----	----	----	----	----	----	----	----
40	2-Nonanone	1072	1070	3.1	0.5	0.2	12.8	0.4	----	0.1	2.7	0.3	4.7	0.09	0.08
41	*p*-Cymenene	1073	1072	----	----	----	----	----	----	0.07	----	----	----	0.03	0.1
42	Enanthylic acid—C_7:0_	1074	1073	----	----	0.09	----	----	----	----	----	0.3	----	----	----
43	Pelargonaldehyde	1085	1084	0.4	----	----	0.5	----	----	----	0.2	----	0.4	----	----
44	2-Nonanol	1089	1087	----	----	----	----	----	----	----	----	----	0.7	----	----
45	Benzenethanol	1093	1091	0.6	3.1	0.1	----	1.6	3.6	0.9	0.4	0.8	0.3	0.7	1.1
46	Undecane ^†^	1100	1100	----	----	----	----	----	----	----	0.2	----	0.2	0.09	0.04
47	Hexahydrobenzoic acid	1110	----	----	----	----	----	----	----	----	----	----	0.4	----	----
48	4-Methyl-benzenemethanol	1117	1122	----	----	0.07	0.2	----	----	----	----	----	----	----	----
49	Camphor ^†^	1121	1125	----	----	----	0.4	----	----	0.03	----	----	----	0.03	0.1
50	(2*E*)-Nonen-1-al	1136	1136	----	----	----	----	----	----	----	0.1	----	0.2	----	0.06
51	1-Nonanol	1164	1162	0.6	----	----	0.03	0.08	0.06	0.1	0.6	----	0.6	0.05	0.07
52	*p*-Cymen-8-ol	1165	1163	----	----	0.02	0.07	----	----	0.08	----	----	0.08	----	0.06
53	Benzoic acid ^†^	1178	1174	1.4	2.5	1.2	0.8	6.0	8.2	5.0	3.7	7.1	4.7	3.5	8.5
54	Ethyl caprylate	1184	1180	1.5	----	----	----	4.1	6.0	7.3	0.7	1.3	0.9	7.6	7.0
55	Caprylic acid—C_8:0_	1187	1180	19.5	34.3	23.6	19.8	15.6	12.2	10.7	5.2	19.6	10.5	11.1	12.4
56	1-Dodecene	1189	1187	0.4	----	----	----	----	----	----	0.4	----	----	----	----
57	(3*Z*)-Dodecene	1194	1195	0.3	----	----	0.4	----	----	----	----	----	----	0.03	----
58	Dodecane ^†^	1200	1200	0.7	0.3	----	0.6	----	----	0.04	0.6	----	1.1	0.6	0.3
59	β-Phenethyl acetate	1235	1233	----	----	----	----	0.2	0.2	0.06	----	----	----	0.08	0.3
60	Isoamyl caproate	1239	1234	----	----	----	----	0.03	----	----	----	0.06	----	----	----
61	δ-Octalactone	1244	1243	0.3	0.07	----	0.09	0.07	0.09	0.06	0.2	0.07	0.2	0.09	0.2
62	^d^ 1,3-Di-tert-butylbenzene	1245	1245	0.1	----	----	----	----	----	----	----	0.05	0.4	----	----
63	(2*Z*)-Decenal	1246	1249	----	----	----	----	----	----	----	----	----	0.2	----	----
64	Decanol	1264	1259	----	----	----	----	----	----	----	0.2	----	0.08	----	----
65	(6*Z*)-Undecen-2-one	1273	1274	----	----	----	0.1	----	----	0.2	----	----	----	----	----
66	Pelargonic acid—C_9:0_	1273	1272	----	0.2	0.4	0.2	0.3	0.3	0.3	0.4	0.3	0.3	0.1	0.3
67	Bornyl acetate	1275	1277	0.4	----	----	----	----	----	----	----	----	----	----	----
68	Propyl octanoate	1280	1277	----	----	----	----	----	----	----	----	----	----	0.05	0.1
69	2-Undecanone	1281	1279	0.9	0.2	0.2	0.9	0.4	0.07	0.1	1.3	0.5	2.5	0.07	0.06
70	Ethyl pelargonate	1285	1282	----	----	0.3	----	0.04	0.05	0.1	----	----	----	0.09	0.05
71	2,6,11-Trimethyldodecane	1286	----	0.1	----	----	----	----	----	----	----	----	0.07	----	----
72	2-Undecanol	1297	1298	----	----	----	----	----	----	0.02	----	----	0.05	----	----
73	Tridecane ^†^	1300	1300	0.1	----	----	----	----	----	----	0.08	----	0.1	0.08	0.03
74	4,6-Dimethyldodecane	1336	----	0.2	----	----	----	----	----	----	----	----	0.04	----	----
75	Isobutyl caprylate	1338	1334	----	----	----	----	----	0.02	0.03	----	----	----	----	----
76	(2*Z*)-Undecenal	1341	1335	----	----	----	----	----	0.02	----	----	0.03	0.04	----	0.04
77	2-Methylundecanal	1358	1350	----	----	----	----	----	----	----	----	----	----	0.06	----
78	Undecanol	1362	1360	----	----	----	----	----	----	0.3	----	----	----	----	----
79	(7*Z*)-Tetradecene	1362	1365	----	----	0.2	1.0	0.3	----	----	4.1	1.0	----	0.4	0.9
80	(2*E*)-Undecen-1-ol *	1374	----	----	----	1.2	5.6	----	----	----	5.0	3.8	0.5	----	3.7
81	Caproleic acid—C_10:1_	1376	1362	1.2	0.8	----	0.8	1.5	0.2	0.1	----	0.7	0.7	1.5	2.0
82	Ethyl 9-decenoate	1377	1370	----	----	----	----	2.3	2.7	4.9	----	----	----	4.9	2.3
83	α-Copaene	1381	1379	0.5	----	----	----	----	----	----	0.2	----	----	----	----
84	Ethyl caprate	1385	1381	0.6	0.8	2.1	0.3	8.6	11.1	11.5	0.7	----	0.4	14.6	4.5
85	Capric acid—C_10:0_	1393	1388	19.7	24.5	45.6	16.1	18.4	13.1	16.4	9.4	22.6	9.5	12.2	11.4
86	Dodecanal	1393	1390	0.5	0.2	----	----	0.06	----	0.05	0.1	----	0.17	0.07	0.03
87	1-Tetradecene	1395	1391	----	----	----	0.4	----	----	----	----	----	----	----	----
88	Tetradecane ^†^	1400	1400	0.4	0.1	----	----	0.2	0.09	0.3	2.2	0.5	0.4	0.3	0.3
89	Cyperene	1402	1404	1.1	----	----	----	0.04	----	0.02	0.2	0.07	0.1	----	----
90	(3*Z*)-Tetradecene	1419	1421	----	----	----	----	----	----	----	0.1	----	----	----	----
91	β-Caryophyllene ^†^	1422	1425	0.3	0.2	0.5	3.7	0.4	0.5	0.9	7.5	1.3	0.6	0.7	1.3
92	γ-Decalactone	1433	1429	----	----	----	----	----	----	----	----	----	0.03	----	0.01
93	α-Guaiene	1440	1441	----	----	----	0.1	----	----	----	0.5	0.2	----	----	----
94	2-Methylbutyl octanoate	1442	1444	----	----	----	----	----	0.03	0.09	----	----	----	----	----
95	α-Humulene	1455	1452	0.2	0.2	0.3	2.9	0.3	0.5	0.7	5.6	1.1	0.4	0.6	1.0
96	δ-Decalactone ^†^	1464	1463	1.0	0.5	0.09	0.3	0.4	0.5	0.3	0.8	0.2	1.3	0.3	0.7
97	Rotundene	1467	1466	0.7	----	----	0.08	0.1	----	----	----	----	----	----	----
98	2,6,10-Trimethyltridecane	1470	1467	0.4	----	----	0.2	0.08	----	0.06	0.3	0.1	0.1	----	0.09
99	Undecanoic acid—C_11:0_	1470	1469	----	0.09	0.3	----	0.09	0.07	0.06	----	0.06	----	0.05	0.07
100	γ-Muurolene	1477	1478	0.2	----	----	0.04	----	----	----	0.09	----	----	----	----
101	Propyl decanoate	1483	1481	----	----	----	----	----	----	----	----	----	----	----	0.06
102	2-Tridecanone	1485	1482	0.2	----	0.5	0.1	0.4	0.07	0.2	0.5	0.3	0.7	0.06	----
103	Ethyl undecanoate	1487	1494	----	----	----	----	0.06	0.07	----	----	----	----	----	----
104	Selinane *	1489	----	----	----	0.1	1.0	0.06	0.07	0.4	3.0	0.5	0.05	0.3	0.5
105	Pentadecane ^†^	1500	1500	0.7	0.2	0.06	0.4	0.1	0.1	0.1	0.3	0.2	0.3	0.2	0.2
106	^d^ BHT	1500	1501	----	0.07	0.03	0.03	----	----	----	----	----	----	----	----
107	^d^ 2,4-Di-tert-butylphenol	1509	1512	0.1	----	0.02	0.09	0.03	0.01	0.02	0.05	0.03	0.05	0.1	0.04
108	γ-Cadinene	1513	1512	----	----	0.02	0.09	trace	0.02	0.04	0.2	0.04	0.02	0.02	0.04
109	*trans*-Calamenene	1517	1515	0.03	----	0.01	0.03	----	0.01	0.02	0.1	0.01	0.02	----	0.03
110	δ-Cadinene	1521	1522	0.05	----	0.03	0.1	trace	0.04	0.06	0.5	0.07	0.03	0.05	0.08
111	Selina-3,7(11)-diene	1534	1535	----	----	----	----	----	----	0.01	0.06	----	----	----	----
112	α-Calacorene	1536	1535	----	----	0.01	0.04	----	----	0.01	0.05	----	----	----	----
113	α-Cadinene	1536	1534	----	----	0.01	0.03	----	----	----	0.03	----	----	----	----
114	Isobutyl caprate	1538	1533	----	----	0.01	----	----	0.03	0.04	----	----	----	----	----
115	2,6,10-Trimethyltetradecane	1554	1557	----	----	----	----	----	----	----	0.06	0.07	0.07	----	0.1
116	(2*E*)-Tridecen-1-ol	1565	1564	----	----	----	----	1.2	0.2	----	0.4	1.2	0.5	----	0.8
117	Lauric acid—C_12:0_	1573	1570	2.2	4.5	8.5	3.1	5.0	4.4	4.7	1.1	2.7	2.3	4.1	2.5
118	(5*Z*)-Dodecenoic acid—C_12:1_	1580	1578	----	----	----	----	0.1	0.2	----	----	----	----	0.4	0.2
119	Ethyl (5*Z*)-dodecenoate	1583	----	----	----	----	----	----	----	0.3	----	----	----	----	----
120	Ethyl laurate	1585	1583	0.1	----	0.1	0.08	3.1	3.5	5.1	0.2	0.1	0.2	5.4	1.5
121	Cetene	1596	1595	0.4	----	----	0.4	----	----	----	0.03	0.04	0.09	----	----
122	Hexadecane ^†^	1600	1600	0.2	----	0.03	0.09	0.04	0.04	0.04	0.08	0.04	0.08	0.05	0.05
123	Myristylaldehyde	1603	1601	0.06	----	0.04	0.03	0.1	0.05	0.04	0.1	0.09	0.1	0.04	0.06
124	Phenylethyl caproate	1620	1619	----	----	----	----	----	----	----	----	0.04	----	----	----
125	Isoamyl n-decanoate	1637	1633	----	----	----	----	----	0.08	0.07	----	----	----	----	----
126	Myristic alcohol	1641	1647	----	----	0.03	0.1	0.06	----	----	0.3	----	----	0.09	0.07
127	2-Methylbutyl decanoate	1643	1647	----	----	----	----	----	----	0.06	----	----	----	----	----
128	γ-Dodecalactone	1646	1645	----	----	----	----	----	----	----	----	----	0.03	----	0.05
129	Tridecanoic acid—C_13:0_	1660	1659	----	----	0.05	----	0.05	----	0.03	----	0.04	----	0.06	0.03
130	δ-Dodecalactone ^†^	1674	1675	0.5	0.3	0.07	0.2	0.4	0.3	----	0.4	0.2	0.6	0.2	0.3
131	13-Methyltetradecanal	1677	1680	----	----	----	----	0.08	----	----	0.1	0.06	0.1	0.04	0.07
132	Ethyl tridecanoate	1688	1689	----	----	----	----	0.04	0.04	0.04	----	----	----	0.1	0.03
133	2-Pentadecanone	1693	1698	0.09	----	0.1	0.01	0.1	0.07	0.1	0.1	0.08	0.2	0.03	0.03
134	Heptadecane ^†^	1700	1700	0.4	0.1	0.03	0.1	0.07	0.04	0.04	0.07	0.04	0.09	0.06	0.05
135	Pentadecanal	1703	1701	----	----	----	----	0.04	----	0.02	0.03	0.03	0.04	----	0.02
136	Myristoleic acid—C_14:1_ *	1752	----	----	0.2	0.4	0.08	0.2	0.1	0.1	----	0.2	0.06	0.2	0.2
137	Myristic acid—C_14:0_	1766	1763	0.4	1.0	2.2	0.8	2.7	1.8	1.5	0.5	1.6	1.1	1.5	1.4
138	Ethyl (9*Z*)-tetradecenoate	1772	1779	----	----	0.1	----	0.3	0.3	0.5	0.07	----	----	1.0	0.4
139	Ethyl myristate	1784	1780	0.05	----	0.04	0.06	1.8	1.6	1.6	0.2	0.1	0.2	2.1	0.7
140	Ethylhexyl salicylate	1790	1805	----	----	----	----	0.09	0.03	----	----	----	----	----	----
141	Octadecene	1796	1797	0.2	0.2	0.04	0.2	0.05	0.07	0.05	0.2	0.08	0.1	0.07	0.08
142	Octadecane ^†^	1800	1800	0.3	0.1	0.03	0.1	0.05	0.06	0.04	0.07	0.04	0.09	0.07	0.05
143	Palmitaldehyde	1803	1807	----	----	----	----	0.1	0.07	0.05	0.1	0.08	0.1	0.03	0.08
144	Phytane	1819	1816	0.09	----	----	----	----	----	----	0.06	----	----	----	----
145	Neophytadiene	1841	1842	----	----	----	0.04	----	0.03	----	0.05	0.02	0.03	----	0.02
146	3,7,11,15-Tetramethylhexadec-2-ene	1851	----	0.8	0.5	0.07	0.3	0.1	0.2	0.09	0.2	0.2	0.3	0.2	0.2
147	Pentadecanoic acid—C_15:0_	1861	1860	----	----	----	----	0.07	----	----	----	----	----	----	----
148	Hexadecanol	1873	1875	----	0.09	0.02	0.3	0.02	----	0.05	0.08	0.06	0.2	0.06	----
149	Ethyl pentadecanoate	1888	1890	----	----	----	----	0.08	0.09	0.05	----	----	----	0.1	0.06
150	δ-Tetradecalactone *	1887	----	0.07	----	0.01	0.03	0.05	----	0.02	0.06	0.04	0.1	----	0.02
151	Palmitoleic acid—C_16:1_	1932	1933	----	----	0.04	----	0.03	----	----	----	0.03	----	----	----
152	(11*Z*)-Hexadecenoic acid—C_16:1_	1952	1953	----	----	----	----	0.07	----	----	----	----	----	----	----
153	Palmitic acid—C_16:0_	1961	1963	0.2	0.2	0.4	0.2	0.6	0.4	0.3	0.2	0.8	0.4	0.3	0.4
154	Ethyl (9*Z*)-hexadecenoate	1958	1955	----	----	----	----	----	0.08	0.09	----	----	----	0.2	0.09
155	Ethyl palmitate	1982	1980	----	----	0.02	0.02	0.4	0.4	0.2	0.07	0.05	0.08	0.2	0.2
156	Isopropyl palmitate	2015	2017	----	----	0.01	0.05	0.07	0.05	----	----	0.03	0.04	0.06	0.05
157	Octadecanol	2073	2074	----	----	----	0.03	----	----	----	----	0.02	0.04	----	----
158	Methyl elaidate	2080	2081	----	----	----	----	0.06	----	----	----	----	----	----	----
159	Methyl stearate	2110	2113	----	----	----	----	0.2	----	----	----	----	----	----	----
160	(9*Z*)-Oleic acid—C_18:1_	2123	2116	----	----	0.04	----	0.06	----	----	----	0.06	----	----	----
161	Ethyl (9*Z*)-oleate	2150	2149	----	----	0.03	----	0.1	0.09	0.06	----	0.09	----	0.1	0.1
162	Ethyl stearate	2180	2181	----	----	----	----	0.02	----	----	----	----	----	----	----
Parcial sum				99.0	99.3	98.4	92.6	97.7	98.2	96.9	84.1	94.2	96.2	96.7	96.7
Unidentified constituents															
Branched hydrocarbons C_11_-C_25_				0.5	0.1	0.4	0.7	----	----	----	0.6	0.7	0.8	0.09	0.04
Branched aliphatic alcohols C_9_-C_11_				----	----	----		0.07	----	----	----	0.2	1.0	----	----
Sesquiterpene hydrocarbons (C_15_H_24_)				----	----	----	0.04	----	----	0.1	0.5	----	----	----	0.03
Sesquiterpene hydrocarbons (C_15_H_26_)				0.4	0.6	0.9	6.0	1.2	1.1	2.1	14.0	3.4	1	1.4	2.7
Branched saturated fatty acids C_9_-C_15_				----	----	0.2	0.2	0.3	0	0.1	0.5	0.3	0.1	0.1	0.2
Branched unsaturated fatty acids C_12_				----	----	----	----	----	----	----	----	0.9	----	----	----
Ethyl ester of unsaturated fatty acids C_12_				----	----	----	----	0.1	0.1	0.2	----	----	----	0.3	0.06
Ethyl ester of branched fatty acids C_13_-C_15_				----	----	----	----	0.2	0.2	0.2	----	----	----	0.6	0.2
Branched aliphatic aldehydes C_15_				----	----	----	----	0.1	0.07	0.1	0.1	0.1	0.09	0.04	0.05
Parcial sum				0.9	0.6	1.4	7.0	2.1	1.6	2.9	15.7	5.7	3.1	2.5	3.3
	
Total (%)				99.9	99.9	99.8	99.5	99.8	99.8	99.8	99.8	99.9	99.3	99.2	100.0

^‡^ Area normalization based on GC-FID analysis and calculated from the average value (mean ± sd) of two measurements. The standard deviation of each identified component ranges between 0.01 (mean values ≤ 1%)–0.5 (mean values ≤ 46.5%); different superscript letters (a–c) show significant differences in chemical compositions (*p* < 0.05). R_I-Cal_: retention index calculated; R_I-it_: retention index of literature; ^†^ compared to the respective certified reference standard (R_t_, R_I_, and mass spectrum); ^d^ contaminant; * tentatively identified. L1: location 1—Cereté 5; L2: location 2—Chinú 2; L3: location 3—Chinú 3; L4: location 4—Chinú 4; L5: location 5—Ciénaga de Oro 4; L6: location 6—Ciénaga de Oro 8; L7: location 7—Sahagún 1; and L8: location 8—Sahagún 3.
