# Supplementary material for: Chemical Profiles of the Volatilome and Fatty Acids of “Suero Costeño” (Fermented Cream)/Raw Milk from Colombia: Promising Criteria for the Autochthonous-Regional Product Identity Designation
Source: Molecules. 2025 Jun 9;30(12):2524. doi: 10.3390/molecules30122524 (PMC12195864; doi:10.3390/molecules30122524)
Supplement: Supplementary file 1 [file molecules-30-02524-s001.zip › molecules-3681207-supplementary.pdf]

Chemical profiles of the volatilome and fatty acids of "suero costeño" (fermented cream)/raw milk from Colombia: promising criteria for the autochthonous-regional product identity designation

Amner Muñoz-Acevedo, Osnaider J. Castillo, Clara Gutiérrez-Castañeda, Mónica Simanca-Sotelo, Beatriz Álvarez-Badel, Alba Durango-Villadiego, Margarita Arteaga-Márquez, Claudia De Paula, Yenis Pastrana-Puche, Ricardo Andrade-Pizarro, Ilba Burbano-Caicedo and Rubén Godoy

Table S1: Result of Shapiro-Wilks test (modified) for fat content yields of RM and SC samples

Variable	Case	Mean	SD	W*	*p* (unilateral D)
RM	8	3.66	0.90	0.86	0.1714
SC	8	7.26	0.95	0.85	0.1503

$p > 0.05$ has no significant differences

Table S2: Result of non-parametric ANOVA using Friedman´s test for fat content yields of RM and SC samples

RM	SC	T^2	*p*
1.00	2.00	$1x10^{30}$	<0.0001

$p < 0.05$ has significant differences

Minimum significant difference between sum of ranks = 0.000

Treatments	Sum (ranks)	Mean (ranks)	Case		
RM	8.00	1.00	8	A	
SC	16.00	2.00	8		B

Means with a common letter are not significantly different ($p > 0.05$).

Table S3: Result of non-parametric ANOVA using Kruskal Wallis´s test for fat content yields of RM and SC samples

Cases	Variable	Means	H	*p*	Variable	Means	H	*p*
L1	RM	2.70	6.92	>0.999	SC	8.2	6.83	>0.999
L2		2.00				7.3		
L3		3.40				7.1		
L4		4.50				7.6		
L5		4.20				8.3		
L6		4.50				7.1		
L7		3.90				7.3		
L8		4.10				5.2		

$p > 0.05$ has no significant differences

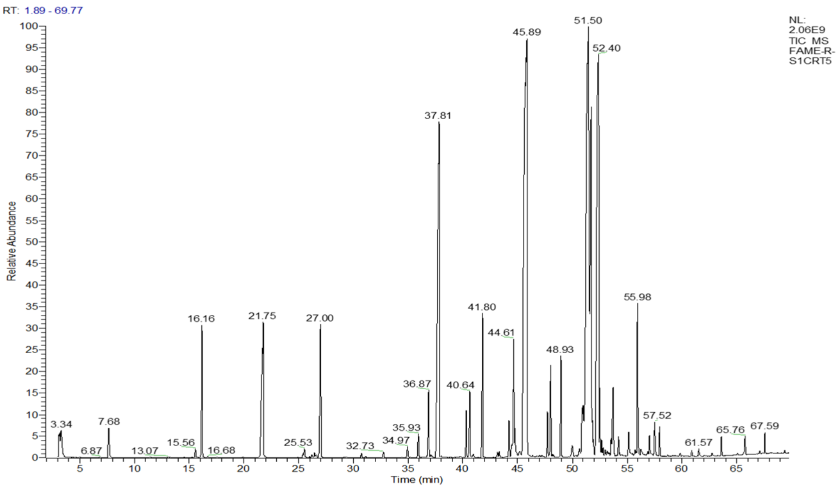

Figure S1: FAME profiles by GC-MS of a suero costeño sample (location 1)

Table S4: Identification and content (>0.4%) of fatty acids in samples of raw milk and suero costeño from eight locations of Córdoba-Colombia

Peak	Fatty acids*	R_{Ical}	R_{Ilit}	L1		L2		L3		L4		L5		L6		L7		L8	
				RM	SC	RM	SC	RM	SC	RM	SC	RM	SC	RM	SC	RM	SC	RM	SC
1	Caproic acid - $C_{6:0}$	896	884	0.2	0.7	1.6	0.3	1.3	0.4	0.6	1.1	1.6	0.9	0.6	0.7	0.2	0.3	1.1	0.7
2	†Caprylic acid - $C_{8:0}$	1091	1083	0.2	0.6	1.0	0.3	0.8	0.6	0.4	0.7	1.0	0.7	0.4	0.7	0.2	0.3	0.5	0.6
3	†Capric acid - $C_{10:0}$	1306	1308	0.7	1.8	2.3	1.5	1.8	2.3	1.5	1.7	2.2	1.9	1.3	2.0	0.9	1.1	1.0	1.4
4	†Lauric acid - $C_{12:0}$	1513	1508	1.6	2.4	2.7	2.9	2.2	3.3	2.8	2.3	2.7	2.5	2.2	2.7	1.7	1.8	1.4	1.9
5	Tridecanoic acid - $C_{13:0}$	1618	1608	0.1	0.1	0.1	0.1	0.1	0.1	0.1	0.1	0.1	0.1	0.1	0.1	0.1	0.1	0.1	0.1
6	iso-Myristic acid - $C_{14:0}$	1688	1686	0.3	0.4	0.4	0.4	0.3	0.3	0.3	0.3	0.3	0.4	0.4	0.4	0.4	0.4	0.4	0.4
7	(9*Z*)-Myristoleic acid - $C_{14:1}$	1708	1715	0.3	1.0	0.8	0.3	0.9	0.9	0.4	0.8	1.3	0.9	1.2	1.3	0.5	0.4	0.9	1.1
**8**	**†Myristic acid - $C_{14:0}$**	**1728**	**1725**	**9.4**	**9.9**	**11.1**	**12.1**	**8.8**	**12.2**	**11.8**	**10.0**	**10.9**	**9.7**	**10.7**	**10.8**	**8.7**	**8.5**	**7.1**	**8.3**
9	13-Methyltetradecanoic acid - $C_{15:0}$	1783	1779	0.6	0.6	0.6	0.6	0.5	0.5	0.5	0.5	0.6	0.5	0.6	0.6	0.7	0.6	0.6	0.6
10	12-Methyltetradecanoic acid - $C_{15:0}$	1790	1782	0.9	1.0	1.0	1.0	1.0	0.7	1.0	0.8	0.9	0.9	1.1	1.0	1.1	1.1	1.1	1.1
11	Pentadecanoic acid - $C_{15:0}$	1819	1820	1.8	2.0	1.5	1.6	1.8	1.6	1.6	1.7	1.5	1.9	1.7	1.7	2.1	2.2	2.1	2.1
12	Iso-Palmitic acid - $C_{16:0}$	1886	1883	0.5	0.5	0.5	0.5	0.5	0.4	0.5	0.4	0.4	0.5	0.5	0.5	0.7	0.7	0.6	0.6
13	(7*Z*)-Palmitoleic acid - $C_{16:1}$	1891	1900	0.3	0.2	0.2	0.2	0.3	0.2	0.2	0.2	0.2	0.2	0.2	0.2	0.1	0.1	0.2	0.2
14	†(9*Z*)-Palmitoleic acid - $C_{16:1}$	1897	1904	0.6	1.6	1.6	0.6	1.5	1.3	0.7	1.4	1.8	1.6	1.9	1.9	1.1	0.9	2.1	2.2
15	(11*Z*)-Palmitoleic acid - $C_{16:1}$	1901	1913	0.9	0.5	0.4	0.6	0.5	0.5	0.8	0.4	0.4	0.6	0.6	0.4	1.0	1.0	0.6	0.5
**16**	**†Palmitic acid - $C_{16:0}$**	**1930**	**1926**	**26.4**	**23.2**	**25.7**	**26.8**	**21.0**	**25.7**	**28.9**	**25.2**	**25.6**	**23.4**	**26.9**	**25.2**	**23.0**	**21.9**	**23.3**	**22.2**
17	14-Methylhexadecanoic acid - $C_{17:0}$	1982	1984	0.5	0.6	0.5	0.5	0.6	0.5	0.6	0.5	0.5	0.6	0.6	0.5	0.6	0.6	0.6	0.6
18	iso-Heptadecanoic acid - $C_{17:0}$	1990	1990	0.9	1.0	0.9	0.8	1.2	1.0	0.9	0.9	0.9	1.1	1.1	1.0	1.0	0.9	1.2	1.2
19	Margaric acid - $C_{17:0}$	2017	2008	1.2	1.2	1.0	1.1	1.2	1.2	1.2	1.1	0.9	1.3	1.1	1.0	1.4	1.4	1.4	1.4
20	†(9*Z*,12*Z*)-Linoleic acid - $C_{18:2}$	2081	2077	1.1	0.6	1.3	0.7	1.1	1.1	0.6	1.2	0.8	0.6	1.0	0.8	0.7	0.6	0.7	0.5
21	†(9*Z*,12*Z*,15*Z*)-Linolenic acid - $C_{18:3}$	2084	2083	0.8	1.0	1.1	0.5	0.8	0.4	0.5	0.8	1.1	0.8	0.5	0.5	tr	0.5	0.4	0.6
**22**	**†(9Z)-Oleic acid - $C_{18:1}$**	**2097**	**2098**	**15.1**	**18.9**	**17.0**	**13.3**	**21.7**	**17.5**	**13.5**	**17.8**	**18.1**	**19.2**	**20.4**	**20.1**	**17.9**	**16.3**	**23.4**	**22.2**
**23**	**(11*Z*)-Vaccenic acid - $C_{18:1}$**	**2099**	**2105**	**4.4**	**2.0**	**1.4**	**4.2**	**3.2**	**2.3**	**4.4**	**1.3**	**1.3**	**2.2**	**2.1**	**1.6**	**4.3**	**5.2**	**1.6**	**1.6**
**24**	**(9*E*)-Elaidic acid - $C_{18:1}$**	**2104**	**2109**	**6.0**	**5.2**	**5.9**	**6.6**	**5.0**	**5.3**	**4.7**	**6.3**	**5.9**	**5.6**	**4.0**	**4.6**	**6.6**	**7.6**	**5.1**	**5.0**
25	(11*E*)-Vaccenic acid - $C_{18:1}$	2107	2115	1.2	1.0	0.3	0.5	0.3	0.3	0.5	0.5	trace	0.7	0.3	0.3	0.7	0.8	0.6	0.6
26	(6*Z*)-Petroselinic acid - $C_{18:1}$	2109	2104	0.8	0.7	0.3	0.4	0.4	0.2	0.3	0.3	0.3	0.5	0.3	0.3	0.5	0.5	0.3	0.5
**27**	**†Stearic acid - $C_{18:0}$**	**2126**	**2124**	**17.1**	**13.7**	**13.1**	**15.9**	**14.2**	**14.2**	**14.9**	**15.6**	**12.0**	**14.3**	**12.6**	**13.4**	**16.9**	**17.3**	**14.9**	**14.7**
28	(9*E*,12*E*)-Linoleaidic acid - $C_{18:2}$	2129	2112	0.7	0.8	0.7	0.4	1.2	0.5	0.3	0.7	1.2	0.9	0.7	0.4	0.9	0.6	0.6	0.7
29	Isomer linoleaidic acid - $C_{18:2}$	2164	----	0.7	0.6	0.3	0.5	0.3	0.4	0.2	0.3	0.3	0.5	0.4	0.4	0.7	0.7	0.5	0.5
30	(9*E*,11*E*)-Linoleaidic acid - $C_{18:2}$	2167	2187	1.4	1.3	1.2	1.1	1.0	0.8	0.9	1.0	1.1	1.2	1.0	1.1	1.4	1.5	1.4	1.4
31	(10*Z*)-Nonadecenoic acid - $C_{19:1}$	2184	2185	0.2	0.3	0.3	0.2	0.3	0.2	0.3	0.2	0.2	0.3	0.2	0.2	0.3	0.3	0.3	0.3
32	Nonadecanoic acid - $C_{19:0}$	2215	2211	0.3	0.4	0.4	0.3	0.3	0.3	0.2	0.3	0.2	0.4	0.3	0.3	0.3	0.3	0.4	0.4
33	(11*Z*)-Eicosenoic acid - $C_{20:1}$	2282	2283	0.2	0.3	0.2	0.2	0.3	0.2	0.2	0.2	0.3	0.3	0.3	0.3	0.2	0.2	0.3	0.3
34	†Arachid acid - $C_{20:0}$	2314	2310	0.4	0.3	0.3	0.4	0.3	0.3	0.3	0.4	0.3	0.3	0.3	0.3	0.4	0.3	0.4	0.3
35	Heneicosanoic acid - $C_{21:0}$	2417	2410	0.1	0.08	0.07	0.1	0.05	0.06	0.09	0.08	0.07	0.08	0.04	0.04	0.1	0.1	0.08	0.09

36	(7Z,10Z,13Z,16Z,19Z)-Docosapentaenoic acid - $C_{22:5}$	2444	----	0.1	0.1	0.1	0.09	0.1	0.1	0.09	0.1	0.09	0.1	0.1	0.1	0.07	0.08	0.09	0.1
37	Behenoic acid - $C_{22:0}$	2524	2511	0.2	0.2	0.2	0.2	0.1	0.2	0.2	0.2	0.2	0.2	0.1	0.2	0.2	0.2	0.2	0.2
38	Tricosanoic acid - $C_{23:0}$	2610	2612	0.2	0.3	0.3	0.3	0.2	0.1	0.3	0.2	0.2	0.2	0.2	0.2	0.2	0.2	0.2	0.1
39	Lignoeric acid - $C_{24:0}$	2710	2713	0.2	0.2	0.2	0.2	0.2	0.12	0.2	0.2	0.1	0.1	0.1	0.2	0.2	0.2	0.2	0.2
40	Pentacosanoic acid - $C_{25:0}$	2808	2813	0.03	0.04	trace	trace	0.03	0.02	0.03	0.03	0.03	0.02	0.03	0.03	0.03	0.03	0.02	0.04
	**Total (%)**			99.6	99.4	99.7	99.3	99.3	99.6	99.2	99.8	99.4	99.3	99.4	99.3	99.5	99.3	99.6	99.4
	**Type of FA**																		
	SFA			63.9	61.6	65.5	68.1	58.8	66.2	69.2	64.7	63.4	62.2	63.1	63.6	61.3	60.8	59.1	59.6
	MUFA			30.7	32.7	29.3	27.7	35.4	29.8	26.9	30.5	31.0	32.7	32.4	32.2	34.0	34.1	36.2	35.5
	PUFA			5.0	4.9	4.8	3.5	4.9	3.6	2.9	4.6	5.0	4.3	3.9	3.6	4.2	4.4	4.1	4.2
	**Type of omega**																		
	n-3 FA			1.0	1.2	1.2	0.7	1.0	0.6	0.7	1.0	1.2	1.0	0.7	0.6	0.2	0.7	0.5	0.8
	n-6 FA			1.1	0.9	1.5	0.7	1.4	1.3	0.6	1.5	1.2	0.8	1.1	1.1	0.7	0.6	0.9	0.8
	n-9 FA			21.7	25.0	23.6	20.5	27.6	23.5	18.9	24.9	24.8	25.6	25.1	25.5	25.2	24.5	29.5	28.1
	**Indices**																		
	AI			2.0	1.9	2.3	2.7	1.5	2.4	2.8	2.0	2.2	1.9	2.1	2.1	1.7	1.6	1.4	1.6
	TI			2.4	2.2	2.5	3.2	2.0	2.9	3.3	2.6	2.3	2.3	2.6	2.6	2.7	2.3	2.2	2.1

*Fatty acids identified as methyl ester derivatives by GC-FID/MSD [area normalization based on GC-FID analysis and it resulted from the average value (mean ± sd) of two measurements. The standard deviation of each identified component ranged between 0.01 (mean values ≤ 1%) – 0.3 (mean values ≤ 28.9%)], †Identified by R_t, R_I and mass spectrum of the FAME present in the Mix C_8-C_{24} certified standard (Table S1), R_{Ical}: Retention indices calculated and determined by GC-FID, R_{Ilit}: Retention indices literature, L1: Location 1-Cereté 5, L2: Location 2-Chinú 2, L3: Location 3-Chinú 3, L4: Location 4-Chinú 4, L5: Location 5-Ciénaga de Oro 4, L6: Location 6-Ciénaga de Oro 8, L7: Location 7-Sahagún 1, L8: Location 8-Sahagún 3, RM: Raw milk, SC: Suero costeño, tr: Traces, SFA: Saturated fatty acids, MUFA: Monounsaturated fatty acids, PUFA: Polyunsaturated fatty acids, AI: Atherogenic indices, TI: Thrombogenic indices.

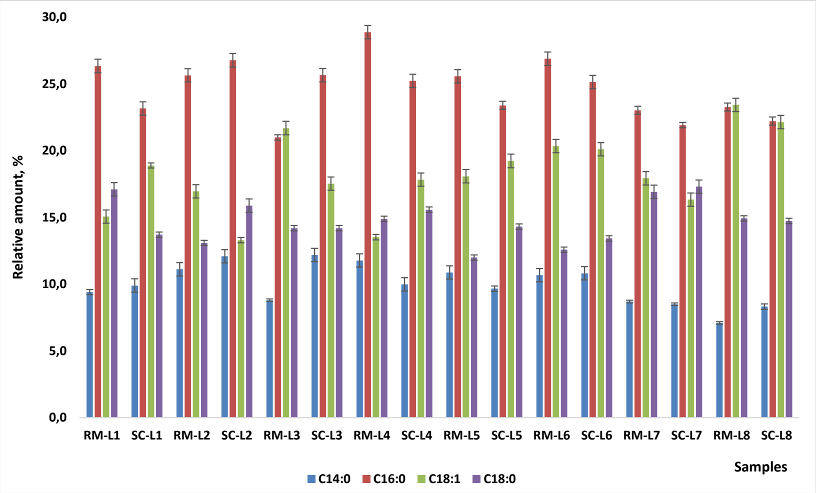

Figure S2: Distribution of the four main FA that constituted the RM and SC samples from the eight locations studied.

Table S5: Result of Shapiro-Wilks test (modified) for major fatty acids of RM and SC samples

Variable	Case	Mean	SD	W*	p (unilateral D)
			RM		
$C_{14:0}$	8	9.81	1.58	0.93	0.6200
$C_{16:0}$	8	25.10	2.52	0.96	0.8761
$C_{18:1}$	8	18.39	3.32	0.96	0.8716
$C_{18:0}$	8	14.46	1.88	0.91	0.4275
			SC		
$C_{14:0}$	8	10.19	1.46	0.89	0.3497
$C_{16:0}$	8	24.20	1.77	0.91	0.4556
$C_{18:1}$	8	18.16	2.66	0.99	0.9850
$C_{18:0}$	8	14.89	1.30	0.92	0.5759

$p > 0.05$ has no significant differences

Table S6: Result of non-parametric ANOVA using Friedman´s test for major fatty acids of RM and SC samples

	L1	L2	L3	L4	L5	L6	L7	L8	T^2	p
**RM**	5.00	4.50	3.75	5.63	4.00	5.00	3.75	4.38	0.24	0.9685
**SC**	3.00	5.75	5.25	5.13	4.25	4.88	3.25	4.00	0.46	0.8546

$p > 0.05$ has no significant differences
Minimum significant difference between sum of ranks (RM) = 15.974
Minimum significant difference between sum of ranks (SC) = 15.476

Treatments	Case	Sum (ranks)	Mean (ranks)	n		Case	Sum (ranks)	Mean (ranks)	n	
L1	RM	20.00	5.00	4	A	SC	14.00	3.50	4	A
L2		18.00	4.50	4	A		23.00	5.75	4	A
L3		15.00	3.75	4	A		21.00	5.25	4	A
L4		22.50	5.63	4	A		20.50	5.13	4	A
L5		16.00	4.00	4	A		17.00	4.25	4	A
L6		20.00	5.00	4	A		19.50	4.88	4	A
L7		15.00	3.75	4	A		13.00	3.25	4	A
L8		17.50	4.38	4	A		16.00	4.00	4	A

Means with a common letter are not significantly different ($p > 0.05$).

Table S7: Result of non-parametric ANOVA using Kruskal Wallis´s test for major fatty acids of RM and SC samples

Variable	Case	Means	H	p
		RM		
$C_{14:0}$	8	7.10-11.80	7.00	>0.9999
$C_{16:0}$	8	21.00-28.90	7.00	>0.9999
$C_{18:1}$	8	13.50-23.40	7.00	>0.9999
$C_{18:0}$	8	12.00-17.10	6.92	>0.9999

SC				
$C_{14:0}$	8	8.30-12.20	7.00	>0.9999
$C_{16:0}$	8	21.90-26.80	6.92	>0.9999
$C_{18:1}$	8	13.30-22.20	7.00	>0.9999
$C_{18:0}$	8	13.40-17.30	7.00	>0.9999

p > 0.05 has no significant differences

Table S8: Result of Shapiro-Wilks test (modified) for the content of fatty acids by compound families of RM and SC samples

Variable	Case	Mean	SD	W*	P (unilateral D)
SFA	16	63.19	3.05	0.94	0.6094
MUFA	16	31.94	2.74	0.95	0.6500
PUFA	16	4.24	0.62	0.91	0.2685
n-3	16	0.82	0.28	0.91	0.2767
n-6	16	1.01	0.31	0.89	0.1209
n-9	16	23.92	3.82	0.91	0.2604

p > 0.05 has no significant differences

Table S9: Result of non-parametric ANOVA using Friedman´s test for the content of fatty acids by compound families of RM and SC samples

	L1	L2	L3	L4	L5	L6	L7	L8	T^2	*p*
**RM**	4.75	5.42	5.58	2.58	5.67	4.00	3.67	4.33	1.21	0.3251
**SC**	5.08	2.92	3.83	5.92	5.25	4.17	3.92	4.92	0.93	0.4986

p > 0.05 has no significant differences
Minimum significant difference between sum of ranks (RM) = 16.853
Minimum significant difference between sum of ranks (SC) = 17.229

Table S10: Result of non-parametric ANOVA using Kruskal Wallis´s test for the content of fatty acids by compound families of RM and SC samples

Variable	Case	Means	H	*p*
SFA	16	58.80-69.20	15.00	0.4514
MUFA	16	26.90-36.20	14.98	0.4514
PUFA	16	2.90-5.00	14.91	0.4514
n-3	16	0.20-1.20	14.45	0.4514
n-6	16	0.60-1.50	14.80	0.4514
n-9	16	18.90-29.50	15.00	0.4514

p > 0.05 has no significant differences

Table S11: Result of Shapiro-Wilks test (modified) for AI and TI values of RM and SC samples

Variable	Case	Mean	SD	W*	P (unilateral D)
AI	16	2.02	0.41	0.94	0.5202
TI	16	2.53	0.37	0.92	0.3186

p > 0.05 has no significant differences

Table S12: Result of non-parametric ANOVA using Friedman´s test for AI and TI values of RM and SC samples

	L1	L2	L3	L4	L5	L6	L7	L8	T^2	*p*
**RM**	5.25	5.50	1.5	8.0	4.50	5.00	4.75	1.50	3.34	0.0672
**SC**	2.75	8.00	7.00	5.25	3.50	5.75	2.50	1.22	22.43	0.000.

p < 0.05 has significant differences
Minimum significant difference between sum of ranks (RM) = 7.843
Minimum significant difference between sum of ranks (SC) = 3.344

Table S13: Result of non-parametric ANOVA using Kruskal Wallis´s test for AI and TI values of RM and SC samples

Variable	Case	Means	H	*p*
AI	16	1.40-2.80	14.91	0.4514
TI	16	2.00-3.30	14.78	0.4514

p > 0.05 has no significant differences

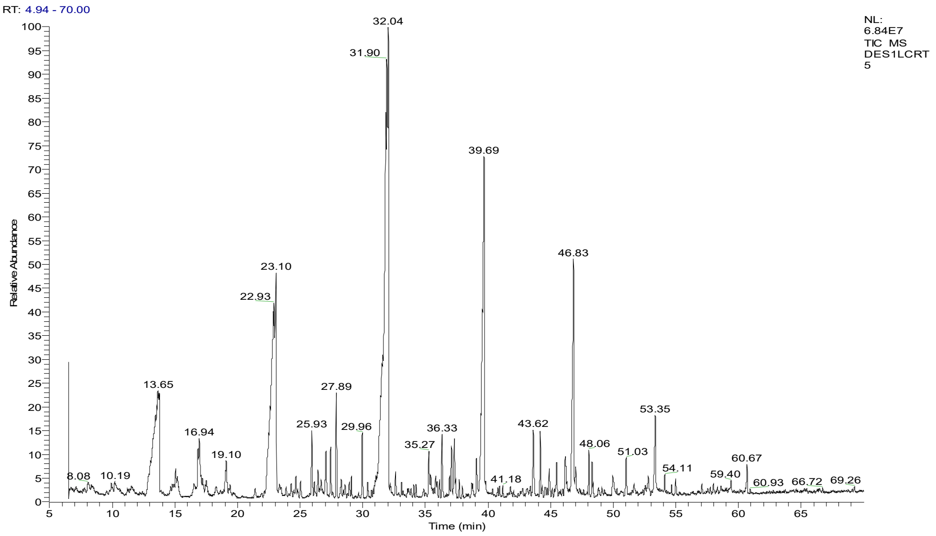

(A)

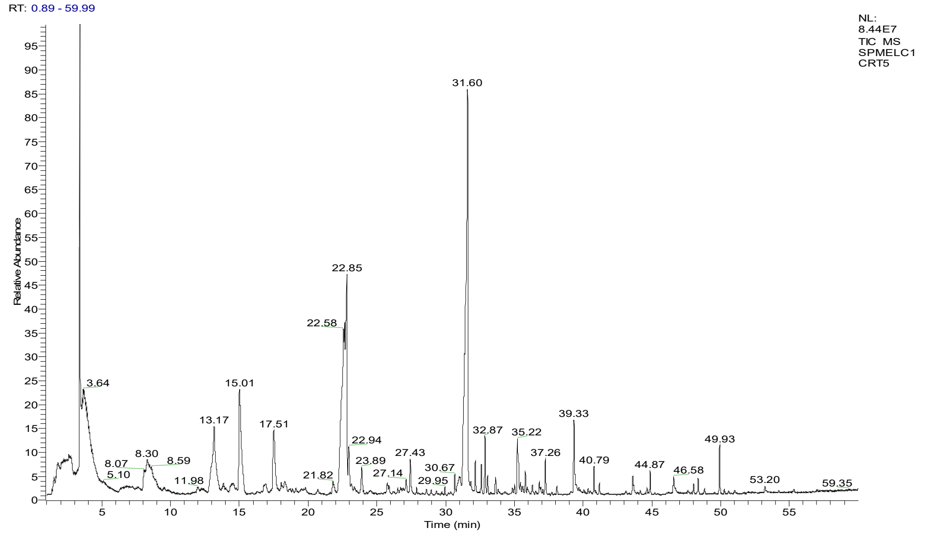

(B)

Figure S3: Volatile profiles by GC-MS of the raw milk (location 1) by (A)SDE and (B) HS-SPME

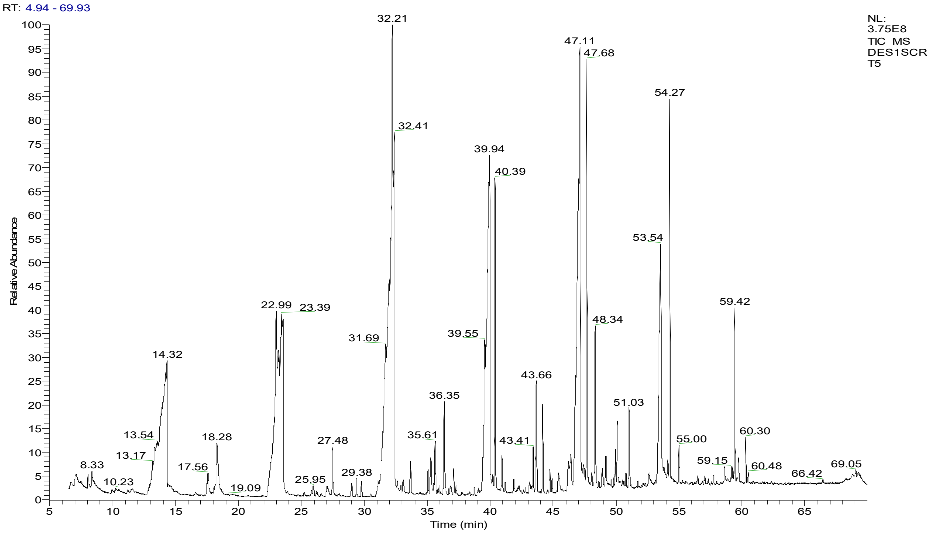

(A)

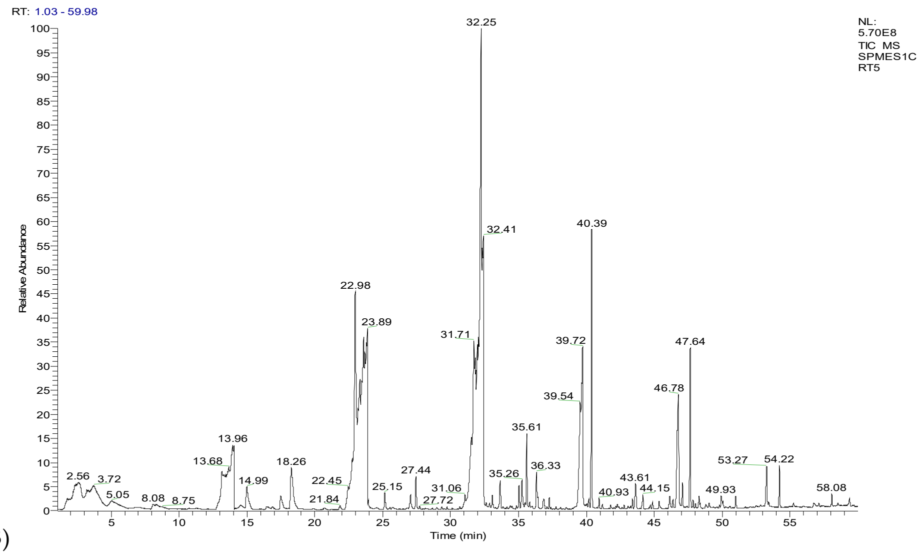

(B)

Figure S4: Volatile profiles by GC-MS of the suero costeño (location 1) by SDE (A) and HS-SPME (B)

Table S14: Result of non-parametric ANOVA using Kruskal Wallis´s test for the volatile fractions by SDE and HS-SPME of raw milk samples

Variable	Case	H	p	Variable	Case	H	p
SDE-L1	18	16.93	0.4544	SPME-L1	18	16.98	0.4544
SDE-L3	18	16.93	0.4544	SPME-L3	18	17.00	0.4544
SDE-L4	18	16.98	0.4544	SPME-L4	18	17.00	0.4544
SDE-L7	16	16.91	0.4544	SPME-L7	16	17.00	0.4544

$p > 0.05$ has no significant differences

Table S15: Result of non-parametric ANOVA using Friedman´s test for the volatile fractions by SDE and HS-SPME of raw milk samples

Technique	L1	L3	L4	L7	T^2	*p*
**SDE**	2.86	2.92	2.31	1.92	2.95	0.0413
**HS-SPME**	3.11	2.78	1.69	2.42	4.90	0.0045

$p < 0.05$ has significant differences
Minimum significant difference between sum of ranks = 14.185
Minimum significant difference between sum of ranks = 14.019

Treatments	Sum (ranks)	Mean (ranks)	Case		
SDE-L1	34.50	1.92	18	A	
SDE-L3	41.50	2.31	18	A	B
SDE-L4	51.50	2.86	18		B
SDE-L7	52.50	2.92	18		B

Means with a common letter are not significantly different ($p > 0.05$).

Treatments	Sum (ranks)	Mean (ranks)	Case		
SPME-L1	30.50	1.69	18	A	
SPME-L3	43.50	2.42	18	A	B
SPME-L4	50.00	2.78	18		B
SPME-L7	56.00	3.11	18		B

Means with a common letter are not significantly different ($p > 0.05$).

Table S16: Result of non-parametric ANOVA using Friedman´s test comparing the chemical composition (average relative amount) of volatile fractions by SDE and HS-SPME of raw milk samples

SDE	HS-SPME	T^2	*p*
1.44	1.56	0.21	0.6507

$p > 0.05$ has no significant differences
Minimum significant difference between sum of ranks = 9.154

Treatments	Sum (ranks)	Mean (ranks)	Case	
SDE	26.00	1.44	18	A
HS-SPME	28.00	1.56	18	A

Means with a common letter are not significantly different (p > 0.05).

Table S17: Result of non-parametric ANOVA using Kruskal Wallis´s test comparing the chemical composition (average relative amount) of volatile fractions by SDE and HS-SPME of raw milk samples

Variable	Case	H	p	Variable	Case	H	p
SDE	18	16.96	0.4544	HS-SPME	18	17.00	0.4544

p > 0.05 has no significant differences

Table S18: Result of non-parametric ANOVA using Friedman´s test for the volatile fractions by SDE and HS-SPME of the suero costeño samples

	L1	L2	L3	L4	L5	L6	L7	L8	T^2	*p*
**SDE**	4.37	3.97	3.47	5.13	4.05	5.55	4.89	4.55	1.55	0.1554
**SPME**	4.43	3.74	3.45	4.81	4.12	6.10	4.33	5.02	2.89	0.0075

p < 0.05 has significant differences
Minimum significant difference between sum of ranks = 28.906
Minimum significant difference between sum of ranks = 28.513

Treatments	Sum (ranks)	Mean (ranks)	Case			
SDE-L1	83.00	4.37	19	A	B	C
SDE-L2	75.50	3.97	19	A	B	
SDE-L3	66.00	3.47	19	A		
SDE-L4	97.50	5.13	19		B	C
SDE-L5	77.00	4.05	19	A	B	C
SDE-L6	105.50	5.55	19			C
SDE-L7	93.00	4.89	19	A	B	C
SDE-L8	86.50	4.55	19	A	B	C

Means with a common letter are not significantly different (p > 0.05).

Treatments	Sum (ranks)	Mean (ranks)	Case						
SPME-L1	93.00	4.43	21	A	B	C	D	E	
SPME-L2	78.50	3.74	21	A	B				
SPME-L3	72.50	3.45	21	A					
SPME-L4	101.00	4.81	21	A	B	C	D	E	F
SPME-L5	86.50	4.12	21	A	B	C			
SPME-L6	128.00	6.10	21						F
SPME-L7	91.00	4.33	21	A	B	C	D		
SPME-L8	105.50	5.02	21		B	C	D	E	F

Means with a common letter are not significantly different (p > 0.05).

Table S19: Result of non-parametric ANOVA using Kruskal Wallis´s test for the volatile fractions by SDE and HS-SPME of suero costeño samples

Variable	Case	H	p	Variable	Case	H	p
SDE-L1	19	17.98	0.4557	SPME-L1	21	19.94	0.4579
SDE-L2	19	17.98	0.4557	SPME-L2	21	19.94	0.4579
SDE-L3	19	17.98	0.4557	SPME-L3	21	19.94	0.4579
SDE-L4	19	17.97	0.4557	SPME-L4	21	19.74	0.4579
SDE-L5	19	17.92	0.4557	SPME-L5	21	19.87	0.4579
SDE-L6	19	17.98	0.4557	SPME-L6	21	19.99	0.4579
SDE-L7	19	17.98	0.4557	SPME-L7	21	19.95	0.4579
SDE-L8	19	18.00	0.4557	SPME-L8	21	19.95	0.4579

p > 0.05 has no significant differences

Table S20: Sampling places (eight) and manufacturing process of SC in four municipalities of Córdoba (Colombia).

Location (L) town	Sample	Raw material – Process
L1- Cereté	CRT5	Milk fat/Spontaneous fermentation (3 day) – P1
L2 - Chinú	CH2	Milk/Spontaneous fermentation (1 day) – P2
L3 - Chinú	CH3	Boiled milk/Spontaneous fermentation (whey starter) – P3
L4 - Chinú	CH4	Cooked whey residue from cheese + commercial milk cream – P4
L5 - Ciénaga de oro	CDO4	Milk/Fermentation using rennet – P5
L6 - Ciénaga de oro	CDO8	Cooked whey residue from cheese + salting whey – P4
L7 - Sahagún	SH1	Boiled milk/Fermentation using whey starter (1 day) – P3
L8 - Sahagún	SH3	Milk/Spontaneous fermentation (1 day, in "totuma") – P2

P: Process related to the method for obtaining suero costeño. "Totuma" is an artisanal reservoir (for liquids or solids) made from a plant fruit (Crescentia cujete)

Table S21: F Test for equality of variances comparing volatiles between raw milk and suero costeño

Variable	Var(1)	Var(2)	F	p
SCFA	27.83	47.45	0.59	0.486
FSFA	294.81	9641.13	0.03	0.0001
FUFA	2.81	1.03	2.73	0.0978
ALIP-ALD	0.33	13.91	0.02	<0.0001
ALIP-ALC	8.77	14.85	0.59	0.4923
MET-ALK-KET	40.8	27.21	1.5	0.4817
MET-ALC-KET	0.0037	1.12	0.0033	<0.0001
AR-ALD	0.2	0.03	5.67	0.0048
AR-ALC	1.02	0.73	1.38	0.5624
AR-ACID	0.41	6.52	0.06	0.0011
EST	1.65	10022.87	0.00016	<0.0001
LACT	0.65	0.88	0.74	0.7091
MONOT	5	2.94	1.7	0.3672
SESQ	22.2	63.09	0.35	0.1675
LHC	2.54	7.4	0.34	0.1579
BHC	61.04	16.43	3.71	0.0313
AR-HC	1.97	0.93	2.13	0.2076
Group 1	Raw milk			
Group 2	Suero costeño			
n(1)	8			
n(2)	16			

$p < 0.05$ has significant differences

Table S22: Chemical composition of volatile fractions by SDE/GC-FID/MSD of raw milk and "suero costeño" samples classified according to the type of compound identified

Type of compound	Raw Milk				Suero Costeño							
	L1	L3	L4	L7	L1	L2	L3	L4	L5	L6	L7	L8
**SCFA**	9.01	15.3	5.89	0.77	9.48	7.24	4.80	1.48	28.5	7.66	6.39	12.3
**FSFA**	56.4	42.9	70.5	81.6	52.8	53.2	42.6	22.0	53.7	17.9	41.5	37.5
**FUFA**	4.03	3.92	4.42	3.46	0.99	1.03	1.32	1.32	3.08	3.18	0.74	2.97
**ALIP-ALD**	1.21	1.64	0.34	0.13	3.27	0.85	0.68	13.5	1.32	9.30	1.42	1.33
**ALIP-ALC**	0.32	0.14	0.24	0.37	1.66	0.85	1.10	5.29	1.24	7.29	1.65	2.09
**MET-KET**	3.25	2.95	0.14	1.19	3.10	2.09	1.44	11.3	1.69	15.7	2.17	5.81
**AR-ALD**	0.55	----	----	----	0.05	0.11	0.07	----	----	0.38	0.14	0.10
**AR-ALC**	0.33	0.37	0.36	----	1.00	1.83	0.47	0.15	0.79	0.46	1.83	0.41
**AR-ACID**	1.51	1.39	2.40	0.80	2.20	1.40	0.84	1.66	2.33	0.78	2.40	1.92
**EST**	0.34	----	0.30	0.10	20.0	26.1	41.5	5.10	1.17	2.11	28.0	21.1
**LACT**	2.44	1.23	0.37	0.38	1.91	0.99	0.75	3.31	0.20	3.12	0.93	0.73
**MONOT**	0.00	1.53	0.27	0.40	0.00	0.23	0.64	0.49	0.67	0.32	0.55	2.12
**SESQ**	0.00	0.71	0.44	1.15	0.84	1.42	0.84	14.8	2.61	3.36	2.18	8.52
**LHC**	3.81	4.35	1.96	1.53	0.48	0.75	0.69	5.29	1.17	9.05	2.02	1.85
**BHC**	15.2	21.3	11.0	6.99	0.95	1.31	1.91	11.5	1.25	13.7	4.37	0.88

**AR-HC**	0.94	1.17	0.75	0.38	0.90	0.06	0.15	0.65	0.21	4.06	0.32	0.25
**HTC-ALC**	----	----	----	----	0.15	----	----	0.25	----	----	----	0.04
**PhOH**	0.62	0.89	0.61	0.19	0.2	0.13	0.09	0.61	0.07	1.57	0.26	0.06
**HTC-ET**	----	----	----	----	----	0.16	0.14	----	----	----	----	----

SCFA: Short-chain fatty acids (≤ C_6), FSFA: Free saturated fatty acids (≥ C_7), FUFA: Free unsaturated fatty acids, ALIP-ALD: Aliphatic aldehydes, ALIP-ALC: Aliphatic alcohols, MET-KET: Methyl alkyl ketones, AR-ALD: Aromatic aldehydes, AR-ALC: Aromatic alcohols, AR-ACID: Aromatic acids, EST: Esteres, LACT: Lactones, MONOT: Monoterpenoids, SESQ: Sesquiterpenes, LHC: Linear hydrocarbons, BHC: Branched hydrocarbons, AR-HC: Aromatic hydrocarbons, HTC-ALC: Heterocyclic alcohols, PhOH: Phenols, HTC-ET: Heterocyclic ethers.

Table S23: Chemical composition of volatile fractions by HS-SPME/GC-FID/MSD of raw milk and "suero costeño" samples classified according to the type of compound identified

**Type of compound**	**Raw Milk**				**Suero Costeño**							
	**L1**	**L3**	**L4**	**L7**	**L1**	**L2**	**L3**	**L4**	**L5**	**L6**	**L7**	**L8**
**SCFA**	15.4	10.9	4.6	4.8	9.6	9.1	3.28	4.00	18.1	19.2	7.13	9.5
**FSFA**	42.0	64.7	81.2	40.4	43.2	32.3	34.1	17.3	48.3	24.3	29.5	28.8
**FUFA**	1.23	0.95	0.46	0.89	2.00	0.46	0.23	0.00	1.92	0.80	2.00	2.38
**ALIP-ALD**	0.96	0.23	0.04	0.66	0.57	0.21	0.28	1.01	0.39	1.55	0.28	0.41
**ALIP-ALC**	3.43	7.70	2.48	6.19	4.94	7.53	5.73	11.7	6.83	12.2	3.97	10.8
**MET-KET**	10.1	2.00	1.46	18.9	1.85	0.21	0.87	6.79	1.49	9.78	0.25	0.21
**M-A-KET**	0.00	0.11	0.15	0.00	1.40	0.72	1.16	3.35	0.79	2.82	1.33	1.40
**AR-ALD**	0.00	1.23	0.00	0.10	0.15	0.67	0.07	0.00	0.00	0.15	0.40	0.30
**AR-ALC**	0.57	3.10	0.21	0.17	1.63	3.57	0.89	0.42	0.80	0.30	0.73	1.07
**AR-ACID**	1.37	2.49	1.24	0.80	6.02	8.19	5.02	3.68	7.08	4.69	3.50	8.50
**EST**	2.60	0.82	3.45	0.52	23.5	32.3	41.3	2.02	3.53	6.41	40.9	24.3
**LACT**	1.86	0.80	0.17	0.61	0.91	0.85	0.35	1.43	0.43	2.25	0.61	1.20
**MONOT**	5.71	1.71	1.25	5.29	0.86	0.44	0.92	5.46	0.45	5.13	2.62	2.93
**SESQ**	3.37	0.92	1.89	14.2	2.06	2.23	4.39	32.5	6.70	2.31	3.13	5.67
**LHC**	4.17	1.06	0.37	3.98	0.79	0.40	0.61	8.45	1.86	2.80	1.94	1.92
**BHC**	2.47	0.61	0.43	0.95	0.20	0.19	0.15	1.31	1.16	1.42	0.29	0.47
**AR-HC**	4.57	0.36	0.33	0.99	0.15	0.39	0.35	0.37	0.05	0.45	0.44	0.05
**HTC-ALC**	0.00	0.00	0.00	0.00	0.00	0.00	0.00	0.00	0.00	0.36	0.00	0.00
**PhOH**	0.10	0.16	0.07	0.12	0.03	0.01	0.02	0.05	0.03	0.05	0.12	0.04
**AL-ACID**	0.00	0.00	0.00	0.00	0.00	0.00	0.00	0.00	0.00	0.36	0.00	0.00
**DIKET**	0.00	0.00	0.00	0.00	0.00	0.00	0.00	0.00	0.00	2.00	0.00	0.00

SCFA: Short-chain fatty acids (≤ C_6), FSFA: Free saturated fatty acids (≥ C_7), FUFA: Free unsaturated fatty acids, ALIP-ALD: Aliphatic aldehydes, ALIP-ALC: Aliphatic alcohols, MET-KET: Methyl alkyl ketones, AR-ALD: Aromatic aldehydes, AR-ALC: Aromatic alcohols, AR-ACID: Aromatic acids, EST: Esteres, LACT: Lactones, MONOT: Monoterpenoids, SESQ: Sesquiterpenes, LHC: Linear hydrocarbons, BHC: Branched hydrocarbons, AR-HC: Aromatic hydrocarbons, HTC-ALC: Heterocyclic alcohols, PhOH: Phenols, HTC-ET: Heterocyclic ethers, M-A-KET: Methyl alcohol ketones, AL-ACID: aliphatic acids, DIKET: Diketones.

Table S24: Composition of FAME Mix C_8-C_{24} (Ref. 18918. Sigma Aldrich) determined by GC-FID/MSD

**R_t. min**	**FAME**	**R_I**	**Area.%**
7.65	Methyl caprylate - $C_{8:0}$	1108	6.6
15.98	Methyl caprate - $C_{10:0}$	1308	8.5
26.64	Methyl laurate - $C_{12:0}$	1508	9.7
37.28	Methyl myristate - $C_{14:0}$	1708	10.3
44.32	Methyl (9Z)-palmitoleate - $C_{16:1}$	1887	5.5
45.28	Methyl palmitate - $C_{16:0}$	1909	14.0
50.70	Methyl (9Z,12Z)-linoleate - $C_{18:2}$	2077	2.9
50.82	Methyl (9Z,12Z,15Z)-linolenate - $C_{18:3}$	2078	1.3
51.02	Methyl (9Z)-oleate - $C_{18:1}$	2081	5.4
51.99	Methyl stearate - $C_{18:0}$	2109	9.3
57.95	Methyl arachidate - $C_{20:0}$	2310	8.2
62.92	Methyl (13Z)-erucate - $C_{22:1}$	2473	3.9
63.67	Methyl behenate - $C_{22:0}$	2511	7.2
67.74	Methyl lignocerate - $C_{24:0}$	2713	7.1

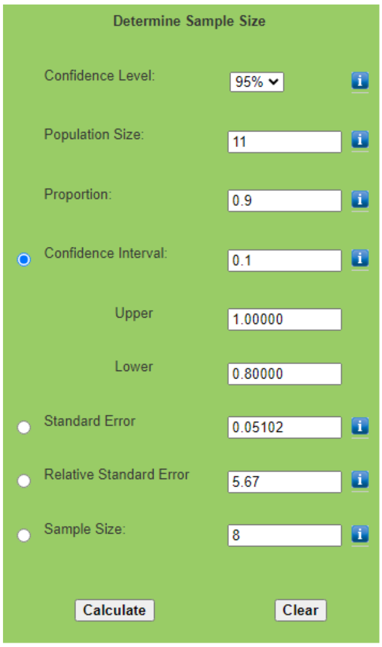

Figure S5: Determination of sample size for SC using sample size calculator (Australian Bureau of Statistics)

Table S25. Selection of samples for chemical analysis based on stratified random sampling

**Process No.**	**Detail of process**	**Total sample No.**	**Sample selection No.**
P1	Milk fat/Spontaneous fermentation (3 day)	1	1
P2	Milk/Spontaneous fermentation (1 day)	3	2
P3	Boiled milk/Spontaneous fermentation (whey starter)	3	2
P4	Cooked whey residue from cheese + commercial milk cream	3	2
P5	Milk/Fermentation using rennet	1	1

Process carried out on small and medium-sized farms where SC samples were taken for this study.
